# Supplementary figures and images for: Metacommunity structure preserves genome diversity in the presence of gene-specific selective sweeps under moderate rates of horizontal gene transfer
Source: PLoS Comput Biol. 2023 Oct 4;19(10):e1011532. doi: 10.1371/journal.pcbi.1011532 (PMC10578598; doi:10.1371/journal.pcbi.1011532)

A

Diversity ( $S$ )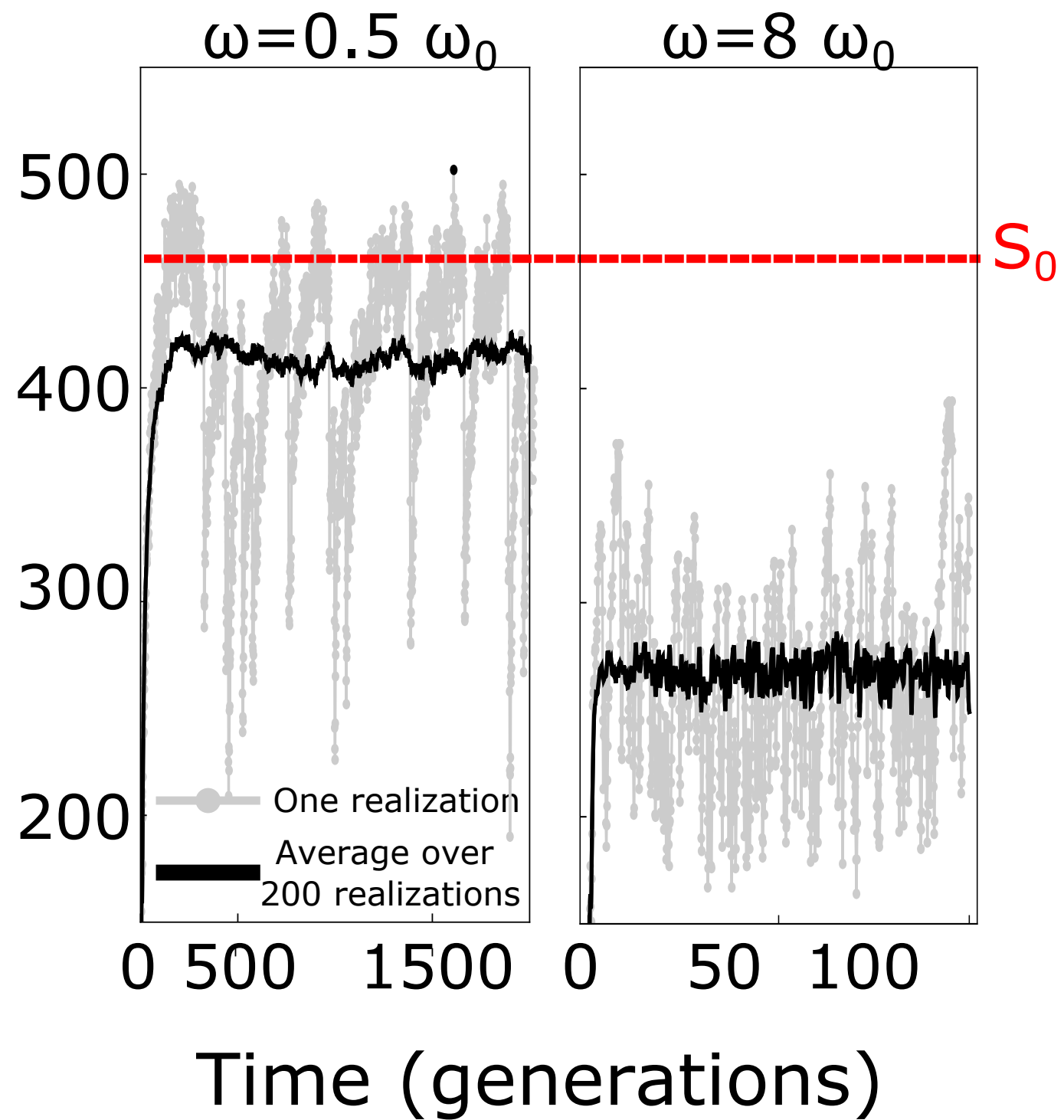

B

Scaled mean  
diversity,  $S_{\text{mean}}/S_0$ 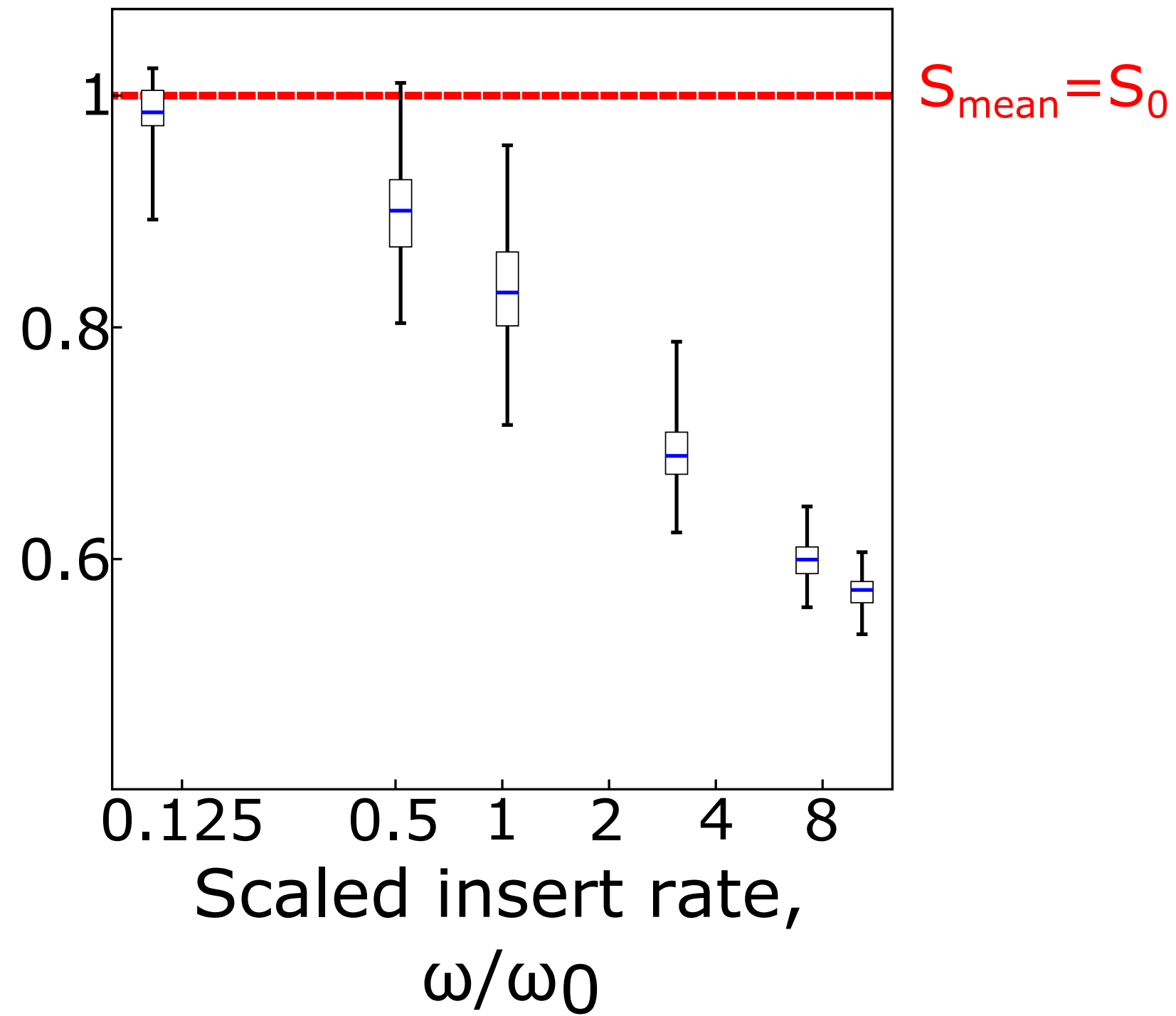

Supplement: S1 Fig — A. We show here two examples of simulations use to investigate the dynamics of the diversity in the same model regime as Fig 4, when beneficial genes emerge stochastically at a constant rate ω (values of ω specified above each plot, in terms of ω0-1=τfix+τeq)). In this case no oscillations are observed, and the dynamics of the diversity is captured by its average value Smean.B. The average diversity (Smean, shown as a box plot) divided by the expected value under neutral biodiversity (S0) shows a monotonic decrease with increasing value of ω/ω0, where ω0-1=τfix+τeq). Other model parameters: M = 10000, ph = 0.1, pm = 0.9. All rates are per patch, per time step unless otherwise specified. (PDF) [file pcbi.1011532.s001.pdf]

A

Stationary Diversity

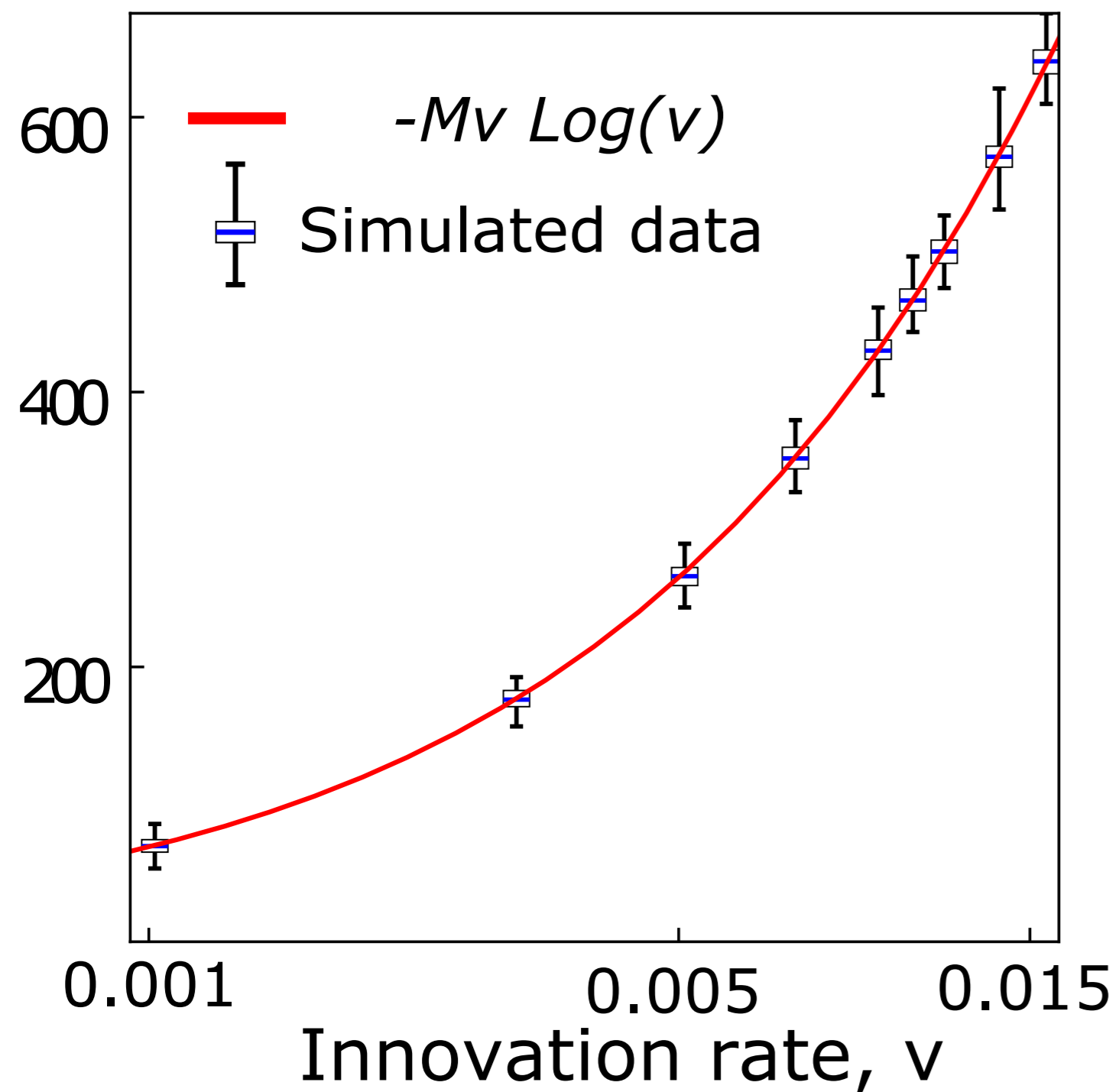

B

Sweep parameter,  $Q_0$ 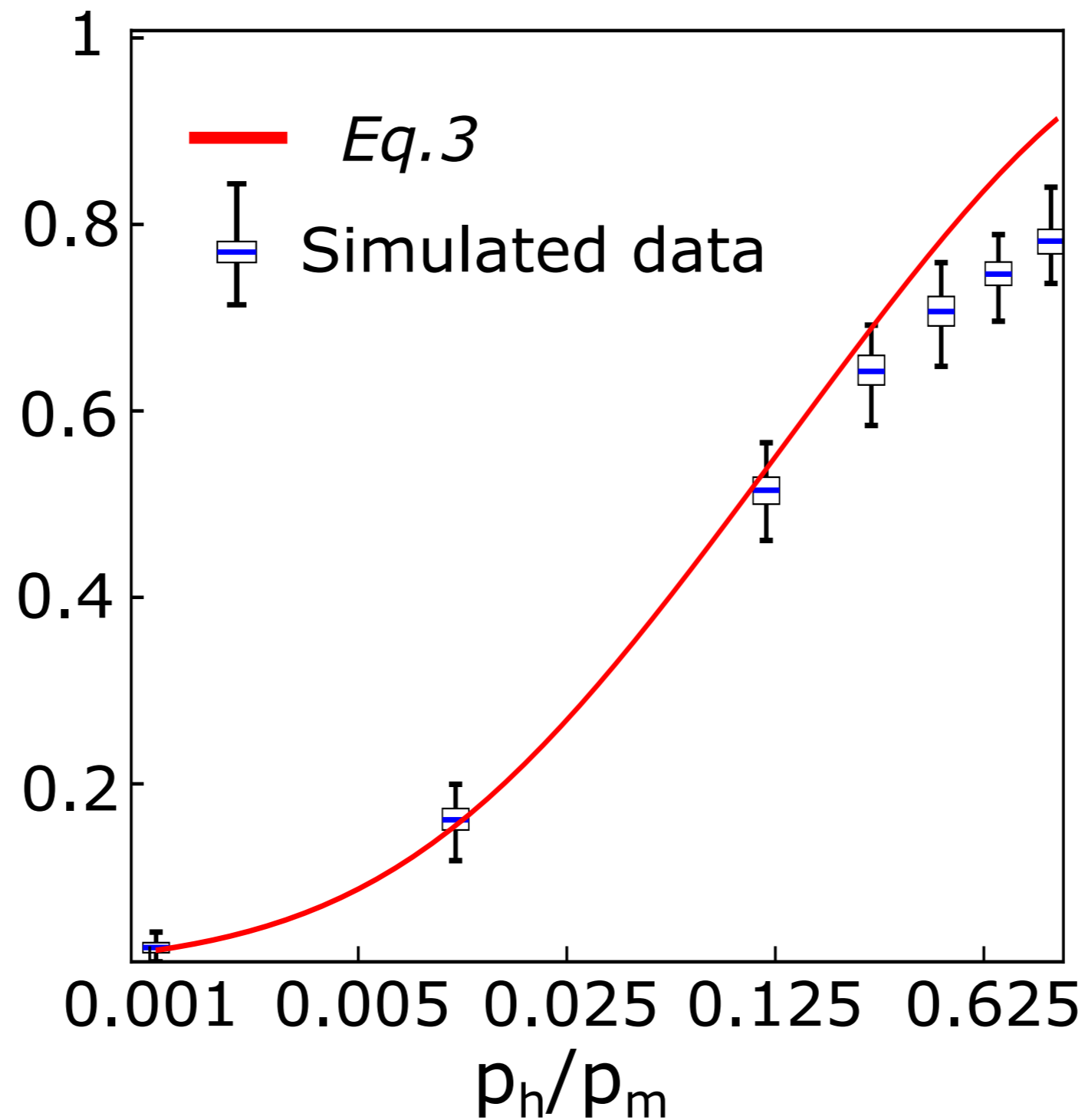

C

Sweep parameter,  $Q_0$ 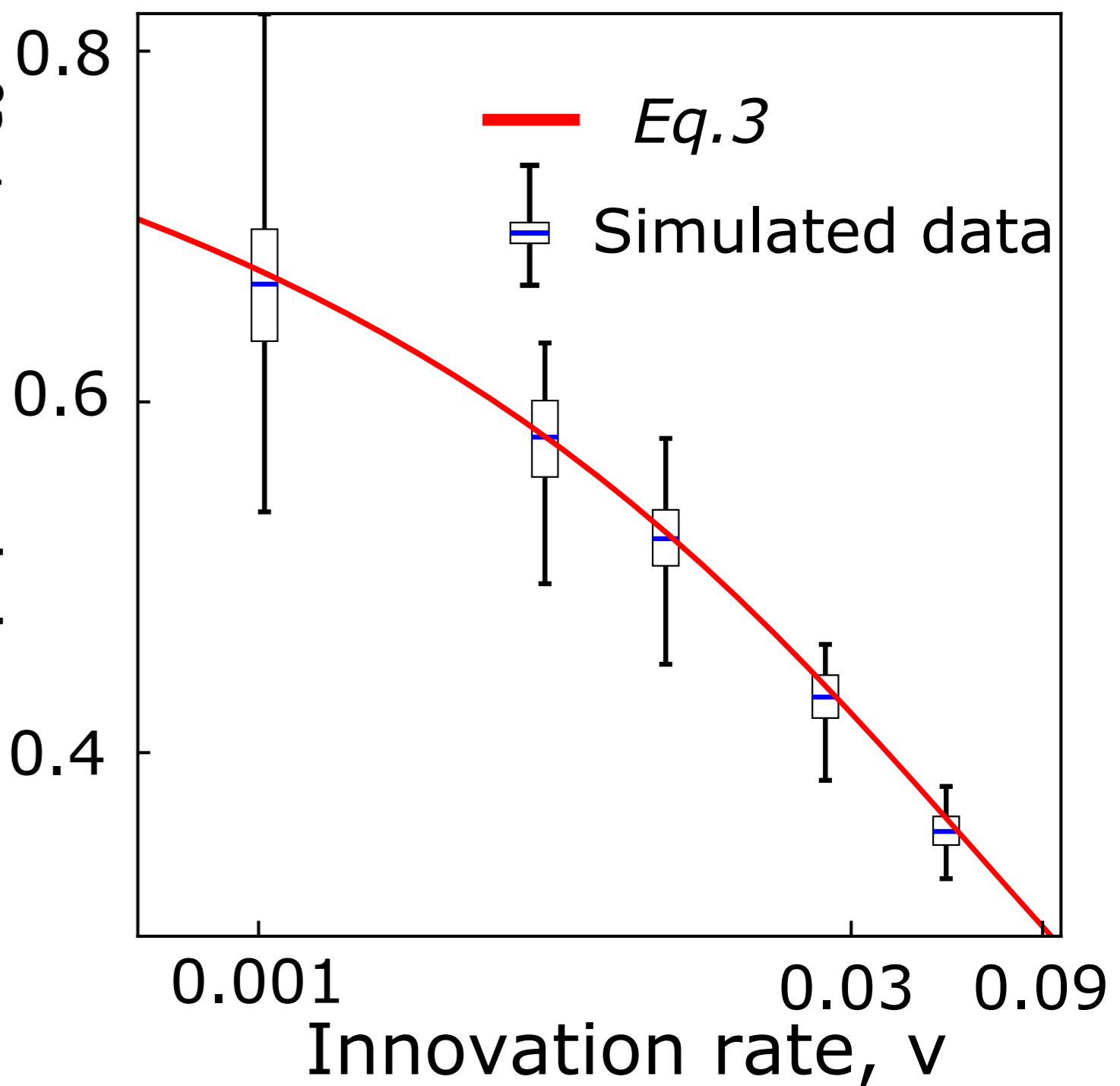

Supplement: S3 Fig — Panels previously displayed in Fig 2C (A), Fig 3C (B) and Fig 3D (C) are presented here on a logarithmic x-scale. (PDF) [file pcbi.1011532.s003.pdf]
