# Supplementary material for: Metacommunity structure preserves genome diversity in the presence of gene-specific selective sweeps under moderate rates of horizontal gene transfer
Source: PLoS Comput Biol. 2023 Oct 4;19(10):e1011532. doi: 10.1371/journal.pcbi.1011532 (PMC10578598; doi:10.1371/journal.pcbi.1011532)
Supplement: S2 Fig — We show here the mean value and the standard deviations of the (i) diversity, defined as the number of distinct species co-existing in the metapopulation (panels A,B and C) and (ii) minimum (Smin)) and maximum (Smax) value of the diversity (panels D,E), evaluated in the dynamical regimes investigated in our model. Simulated data (and corresponding model parameters) used for this analysis are the same one displayed in the main figures, and are specified above each plot. (PDF) [file pcbi.1011532.s002.pdf]

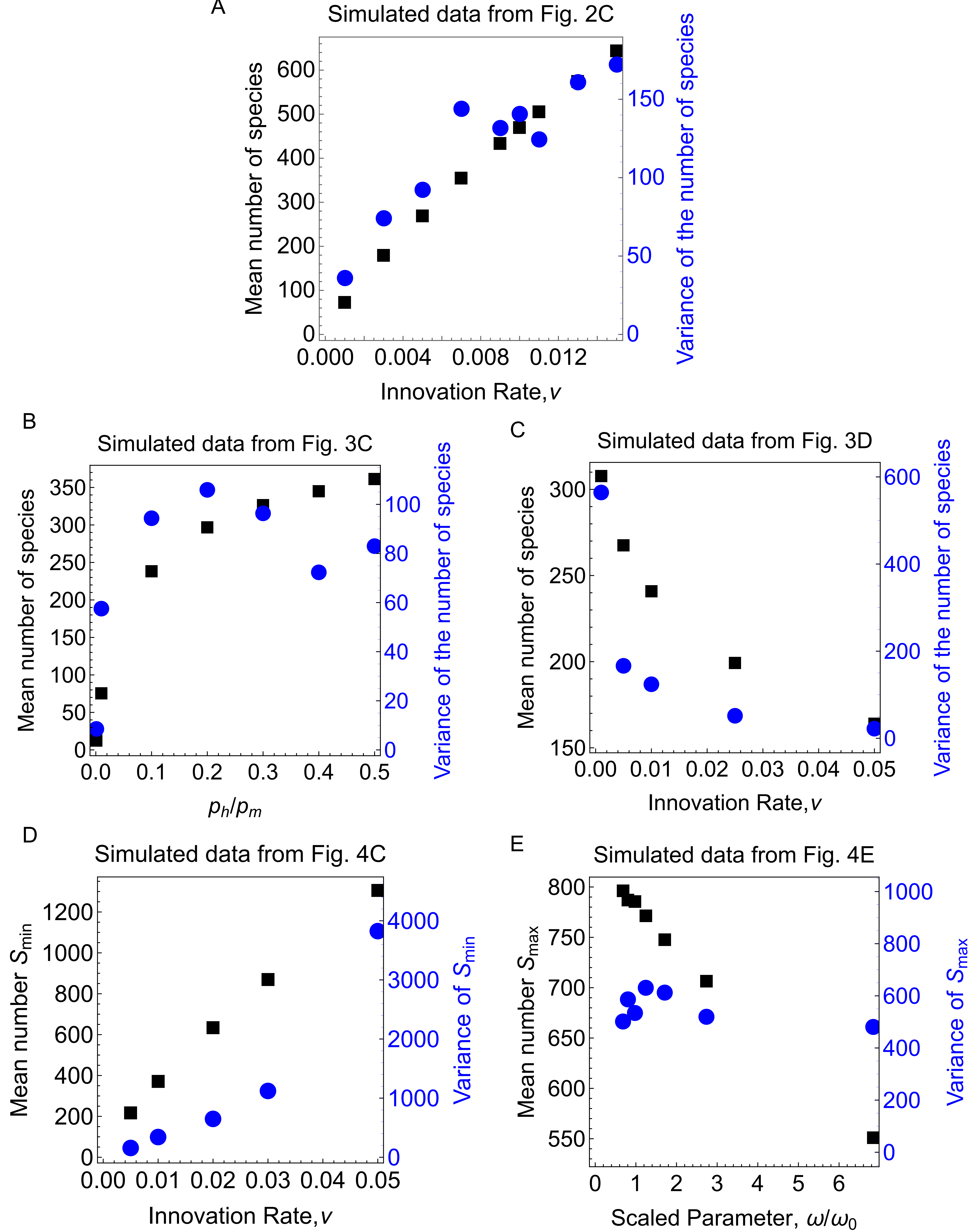
A
Simulated data from Fig. 2C
Mean number of species
Variance of the number of species
0 100 200 300 400 500 600
0 50 100 150
0.000 0.004 0.008 0.012
Innovation Rate, v
B
Simulated data from Fig. 3C
Mean number of species
Variance of the number of species
0 50 100 150 200 250 300 350
0 20 40 60 80 100
0.0 0.1 0.2 0.3 0.4 0.5
p_h/p_m
C
Simulated data from Fig. 3D
Mean number of species
Variance of the number of species
150 200 250 300
0 200 400 600
0.00 0.01 0.02 0.03 0.04 0.05
Innovation Rate, v
D
Simulated data from Fig. 4C
Mean number S_{min}
Variance of S_{min}
0 200 400 600 800 1000 1200
0 1000 2000 3000 4000
0.00 0.01 0.02 0.03 0.04 0.05
Innovation Rate, v
E
Simulated data from Fig. 4E
Mean number S_{max}
Variance of S_{max}
550 600 650 700 750 800
0 200 400 600 800 1000
0 1 2 3 4 5 6
Scaled Parameter, ω/ω_0
