# Supplementary material for: Metacommunity structure preserves genome diversity in the presence of gene-specific selective sweeps under moderate rates of horizontal gene transfer
Source: PLoS Comput Biol. 2023 Oct 4;19(10):e1011532. doi: 10.1371/journal.pcbi.1011532 (PMC10578598; doi:10.1371/journal.pcbi.1011532)
Supplement: S1 Table — This table summarizes the different regimes of the expected value of the biodiversity as a function of two dimensionless variables ω(τfix + τeq), and τfix/τeq, including the three main time scales of the model: (i) the equilibration time of the system (τeq), (ii) the fixation time of the beneficial gene (τfix) and (iii) the time between arrival of beneficial genes (1ω). In each regime we illustrate the (i) expected maximum and minimum biodiversity (Smax and Smin, expressed in terms of the expected neutral value of the diversity S0 (Eq 1) and of the sweep parameter Q0 (Eq 3) and (ii) the Figure showing the corresponding numerical results. (PDF) [file pcbi.1011532.s004.pdf]

Regime	Conditions on time scales	Expected diversity	Figure of reference
Neutral evolution	/	$\langle S\rangle = \langle S_0\rangle$	Fig.2
Time separation (No diversity-maintenance mechanism)	$\tau_{\text{fix}}/\tau_{\text{eq}} \ll 1$	$S_{max} \geq S \geq S_{min}$<br>$\langle S_{max}\rangle = \langle S_0\rangle$<br>$\langle S_{min}\rangle = Q_0 \langle S_0\rangle$	Fig.3
Time scale competition	$\tau_{\text{fix}}/\tau_{\text{eq}} \gtrapprox 1$ and $\omega(\tau_{\text{fix}} + \tau_{\text{eq}}) < 1$	$S_{max} \geq S \geq S_{min}$<br>$\langle S_{max}\rangle = \langle S_0\rangle$<br>$\langle S_{min}\rangle > Q_0 \langle S_0\rangle$	Fig.4 B-C
	$\tau_{\text{fix}}/\tau_{\text{eq}} \gtrapprox 1$ and $\omega(\tau_{\text{fix}} + \tau_{\text{eq}}) > 1$	$S_{max} \geq S \geq S_{min}$<br>$\langle S_{max}\rangle < \langle S_0\rangle$<br>$\langle S_{min}\rangle > Q_0 \langle S_0\rangle$	Fig.4 C-D
