# Supplementary material for: Metacommunity structure preserves genome diversity in the presence of gene-specific selective sweeps under moderate rates of horizontal gene transfer
Source: PLoS Comput Biol. 2023 Oct 4;19(10):e1011532. doi: 10.1371/journal.pcbi.1011532 (PMC10578598; doi:10.1371/journal.pcbi.1011532)
Supplement: Attachment — Submitted filename: comments_to_authors.pdf [file pcbi.1011532.s005.pdf]

Comments to Authors

February 8, 2023

The Authors present a very nice modeling scenario and analysis to explore how selective sweeps and horizontal gene transfer (HGT) affect diversity in a metacommunity (i.e. spatially structured) setting. The main takeaway of the paper is that spatial structure can preserve diversity by making gene-specific, rather than genomewide, sweeps possible, even for low to moderate rates of HGT. Essentially, this occurs because the sweep rate due to vertical transmission is greatly reduced by the spatial structure; the sweep is forced to occur on the timescale of migration, which allows HGT to be "competitive" with this rate. HGT can then allow significant spread of the beneficial gene in a gene-specific fashion, preserving diversity in the metacommunity. This provides a simple and likely ubiquitous mechanism that can explain the prevalence of gene-specific sweeps inferred from data, which has been difficult to explain given empirical estimates of HGT rates.

The Authors develop a very clean explanation for the phenomenon of interest, and provide an impressive mix of analytical and simulation results. I particularly liked the way the Authors built up the full dynamical picture by first considering a background neutral process, then considering a single sweep event, then finally considering how these processes, occurring on several different timescales, interact. Below I offer some suggestions that could improve the manuscript; I separate these into "Major" and "Minor", although "Minor" and "Very Minor" might be more apt.

Major comments

Background and contextualization

I found the paragraph beginning "In the past years" somewhat confusing. First, my reading of the Shapiro et al. paper is that it presents evidence for gene-specific, rather than genomewide sweeps, and also suggests that habitat-specific HGT helps maintain genetic clusters. However, it does not seem to me to provide a hypothesis for the question, "How can gene-specific (or at least not completely genomewide) sweeps occur despite low HGT rates?", which I take to be the central question of the current manuscript. Indeed, Shapiro et al. pose as a major open question "What mechanisms (aside from unrealistically high recombination rates) are responsible for preventing genomewide selective sweeps". Thus, I have a hard time seeing how this constitutes a hypothesized mechanism.

Second, the Authors assert that the mechanism proposed by Niehus et al. requires "a small immigration rate and a high HGT rate". What is the basis for this claim? My understanding of the Niehus paper is that diversity is preserved when migration rates are intermediate (in a specific sense) and that the HGT rates they consider are all "low" (at least relative to the strength of selection, and comparable to empirical estimates for HGT). Can the Authors explain a little more here?

Third, the reference to "basal recombination rate" in the Takeuchi et al. paper is very confusing. The context (basal compared to what?) is not clear here. Additionally, it seems contradictory to say that this hypothesis requires low recombination rates, then state later in the same paragraph that all of these scenarios require high HGT rates.

The Authors should clarify each of these three points. I also might suggest moving some of this content to the Discussion, especially if longer exposition is needed to clarify the confusions I mention above.

Additionally, one major piece of context that seems missing is the classical population genetics perspective on selective sweeps in structured environments. There is a literature on this topic, for example:

Slatkin, M., & Wiehe, T. (1998). Genetic hitch-hiking in a subdivided population. Genetics Research, 71(2), 155-160.

Santiago, E., & Caballero, A. (2005). Variation after a selective sweep in a subdivided population. Genetics, 169(1), 475-483.

Kim, Y., & Maruki, T. (2011). Hitchhiking effect of a beneficial mutation spreading in a subdivided population. Genetics, 189(1), 213-226.

I believe the present manuscript still contributes plenty of novelty, especially since the questions of interest are somewhat different in the bacterial context, and because the mechanics of HGT are somewhat different than sexual recombination. However, the basic intuition that population structure can increase the diversity preserved through a sweep has been recognized before, e.g. in the references above. It would be good to acknowledge this, and perhaps to clearly identify what is new or different in this context.

Unstructured benchmark

The point that metacommunity structure preserves variation compared to a hard sweep (e.g. a sweep with no HGT) is made clear, but it might be helpful for readers to see an explicit comparison to a scenario without spatial structure. For an unstructured population of size MN, how much diversity is preserved? None? My sense is that the resulting sweep would be essentially hard, given the rates considered here, but it would be a nice contrast to explicitly compare against an unstructured benchmark.

Relationship between HGT rates and migration rates

Somewhat related to point above, one aspect of the model framework that confused me a bit was how to think about the relationship between the HGT rate and migration rates. My biological intuition is that they should be linked somehow, such that HGT is inherently less likely when populations are highly separated (e.g. μ_h decreases when μ_m decreases). This is not explicitly enforced, although Eq. 6 implies some relation between these rates. Related to this confusion, what would be the HGT rate in an unstructured population? Should I think of μ_h as the product of μ_m and some baseline HGT rate, which would apply within a local patch? A last point of confusion: The Authors state that this mechanism "is compatible with small HGT rates compared to typical migration time scales", which seems at odds with Eq. 6. Can the Authors clarify all of this?

Probability of extinction

I believe there is a simpler and more accurate way to estimate the probability of extinction (Eq. 21). In the scenario considered here, each patch of a species s will meet one of two fates: it will be replaced by a species with the beneficial gene, or it will receive the beneficial gene through HGT. Both events depend on an interaction with a patch possessing the gene already. The rate at which this interaction happens is essentially irrelevant – once a population of species s is selected to interact with a population that has the gene (at a total rate $p_h + p_m$), it will be replaced with probability $\frac{p_m}{p_h+p_m}$ and HGT happens otherwise. So, the probability that no population of species S survives is just

$$\left(\frac{p_m}{p_h + p_m}\right)^{M_s^0} = \left(1 + \frac{p_h}{p_m}\right)^{-M_s^0}$$

This expression is close to $e^{-M_s^0 \frac{p_h}{p_m}}$ for large M_s^0 and small $\frac{p_h}{p_m}$. One can use this estimate in the sweep parameter, Q, with no modification of the derivation of Q, and the modified Q appears to give a slightly better fit to the simulation data in Fig. 3 (I checked this by eye). Additionally, this argument is much simpler, removing the need for Eqs. 14-20.

Minor comments

- The Authors provide a very nice picture of the temporal dynamics in their scenario. However, the data needed to see these patterns are rare and difficult to produce. Are there any predictions or diagnostic features that could be observed in, for example, spatial patterns or traditional population genetics data (e.g. site frequency spectrum). I realize this is probably beyond the scope of the Manuscript, so I understand if there is not much to be said here, but I do think that even some discussion on this point would substantially strengthen the manuscript.

- The current wording of the title reads awkwardly; I would suggest "A metacommunity scenario preserves diversity in the presence of gene-specific selective sweeps" instead. Additionally, the title seems to mis-state the point slightly – to me as a reader, it's not surprising that gene-specific sweeps preserve diversity, but it is unclear how gene-specific sweeps can compete with genomewide sweeps. Something like "A metacommunity scenario explains gene-specific sweeps under moderate rates of horizontal gene transfer" might be clearer.

- The term "diversity-maintenance mechanism" was confusing at first. By this, I believe the Authors mean some mechanism, such as speciation or immigration of new species, that provides a source of new diversity. I appreciate that the Authors want to emphasize that their results are agnostic to a particular assumption about this mechanism, but I would more clearly explain what is meant by this term when it is first used.

- The use of the term "sweep" for neutral takeover of patches seems likely to cause confusion with selective sweeps. Maybe use an alternate term, such as "neutral takeover" or "drift".

- Instead of "We next ask when diversity decreases only moderately ...", consider a more neutral question, such as "We next ask how much diversity is lost upon the introduction of a beneficial gene, in the absence of diversity-maintenance mechanisms."

- The intuition for the scenario described by Eq. 2 is very clear, but the expression itself is not. The genome sweep time has not been introduced yet, so readers would benefit from slightly more explanation here (and maybe a reference to the supplement). Additionally, I am unsure where the second inequality comes from. What is the metacommunity-wide fixation time? Is this the timescale on which all diversity would be lost if neutral takeover is the only process? Please provide slightly more explanation, and clarify where the timescale MN comes from here.

- "maintenance" is misspelled as "mantainance" on p. 10.

- Just before Eq. 4 there is a tau missing the backslash.

- I was confused by the statement "Inclusion of a diversity-restoring mechanism [...] can increase the minimal observed diversity as well as the maximal one". Should this say "as well as decrease the maximal one"? Or am I missing something?

- Niehus is misspelled both as Niehuis and Niehius at different points. Please double check this.

- Readers might benefit from a reference for the two standard results in the first paragraph of the Methods (the fixation probability and sweep time).

- In the Methods, s is used both for the strength of selection and to denote a species. I would use a different letter for the latter purpose, to avoid any confusion.

- There is an extra label for Eq. 23.
