# Supplementary material for: Metacommunity structure preserves genome diversity in the presence of gene-specific selective sweeps under moderate rates of horizontal gene transfer
Source: PLoS Comput Biol. 2023 Oct 4;19(10):e1011532. doi: 10.1371/journal.pcbi.1011532 (PMC10578598; doi:10.1371/journal.pcbi.1011532)
Supplement: Attachment — Submitted filename: PLOSCompBio_review_PompeiEtAl_2023.pdf [file pcbi.1011532.s007.pdf]

Review of "A Metacommunity Scenario Preserves the Diversity in Presence of Gene-specific Selective Sweeps"
PLOS Computational Biology
February 20, 2023

This manuscript details a theoretical mechanism intended to address the following counterintuitive observation in microbial metagenomic data: despite a widely held assumption that horizontal gene transfer rates remain small such that the population dynamics are predominantly clonal, there appears to be excessive genetic diversity relative to the expectation that selective sweeps of alleles under positive selection are common in microbiota and efficiently purge variation. Specifically, there is an observed excess of diversity proximate to beneficial alleles (genes), which has been argued in the literature to only happen when horizontal gene transfer (HGT) rates are high. The authors address this issue by postulating that the diversity observed in metagenomic samples is not due to intra-population maintenance (or regeneration via mutations), but instead largely due to population structure. Specifically, they model a large number of relatively isolated subpopulations (connected via migratory dynamics), which are inherently pooled together in a metagenomic sample and collectively sequenced; the observed genetic diversity arises due to demographic diversity of the habitat (many local niches where differing species may rapidly fix), rather than due to intrapopulation dynamics that may require invoking mechanisms like ubiquitous recombination, linkage, epistasis, or unrealistically high mutation rates. The mathematical model the authors specify is thus something closer to an ecological model, rather than a panmictic population genetic model. The authors use both population genetic and eco-evolutionary techniques to analytically describe their constructed system and compare this to simulations, which show reasonable agreement to their theoretical analysis.

Overall, I find their proposition reasonable and largely intuitive. The analytics presented are sound (I rederived all presented formulae to confirm this) and the authors decompose relatively complex dynamics inherent to the system via a separation of timescales argument, which I find both convincing and appealing. I have a number of comments on the presentation of their findings, but, qualitatively, I agree with their results and feel they are sufficiently interesting to warrant eventual publication. However, I note that there are a substantial number of simplifying assumptions used to construct their toy model, only some of which are discussed in detail in the text; some of these assumptions are not obviously consistent with the majority of microbial metagenomic systems in my view (e.g., microbial subpopulation sizes small enough to result in subsequent selective sweeps, rather than clonal interference; this impacts the assumption that, at any given time, each subpopulation is generically dominated by a only single lineage/species/strain). More substantial text should be dedicated to the regime of applicability of their model (explicitly linking to measurable population genetic and ecological parameters, where possible), and highlighting the effect of each simplifying assumption on the regimes of validity; this should be done in the beginning of the manuscript, rather than the more minor treatment that currently appears in the Discussion. A deeper overview of the nature of the specific types of metagenomic data the authors address, which should appear in the introduction, is also warranted, as this would better frame the problem and make it more obvious that inter-, rather than intra-population dynamics are applicable to the data used in the literature highlighting this outstanding question (e.g., is the initial observation of excessive diversity only seen in pooled short read sequencing studies?; how does ascertainment of a metagenomic sample affect this proposition—if a bacterial metagenomic sample is generated from a minimally invasive biopsy, how does the potential underrepresentation of the metacommunity affect their results?).

Most importantly, the authors should dedicate much more significant real estate on the notion of 'diversity', as the biodiversity-related meaning of diversity in their theoretical study (the number of species in the metapopulation) is entirely different from common measures of genetic diversity in population genetics that may or may not account for allele frequency-based metrics like heterozygosity (i.e., two-point measures of diversity related to the variance of allele frequency, even in haploid species). In my view, genetic diversity is more naturally applicable to pooled short-read metagenomic sequence data, as the ability to correctly separate and identify distinct 'species'/strains is inherently more difficult unless the read data can be mapped to robust genome-wide references. In particular, the expectation of the number of species in a metacommunity uses no allele frequency information; this measure is analogous to population genetic quantification of the number of segregating sites in a sample, which is well known to be substantially influenced/biased by sample size. In contrast, a two-point measure of diversity

(analogous to heterozygosity in population genetics) would likely provide a more unbiased estimate of pooled sample ‘diversity’, and should be similarly straightforward to measure in simulation data and not much more difficult to treat analytically. In the present context, such a measure corresponds to the probability of finding distinct, rather than the same, species when randomly sampling the metapopulation twice, which is related to the variance, rather than the mean, of the distribution of species in the metacommunity. I am not suggesting that the authors should redo their analysis with such a distinct measure, but a discussion of the robustness of their qualitative findings to differing measures of diversity would reinforce their conclusions, particularly if this can be tied back to the varying ways diversity can be measured in metagenomic sample studies.

General comments:

- The authors need to do significant copy editing prior to publication. There are many syntactical and grammatical mistakes, along with a few spelling errors.
- The introduction highlights a few highly relevant papers but explains their content too briefly. As PLOS Comp. Bio. is an interdisciplinary journal, more detailed presentation of each of these papers would improve the introduction and better frame the problem being addressed. In particular, the mechanisms described in references [6] and [14] are both somewhat realistic, in my view, but are presented very briefly and should be elaborated on and somewhat clarified in the introduction. The authors should also make it very clear that the rate of recombination discussed in this section is not entirely the same as the rate of HGT (e.g., [14] discusses high HGT rates but low per-base recombination); this was unclear to me during my first read through.
- In my experience, presentation of separation of timescales arguments (e.g., adiabatic approximations) can be difficult for the population genetic/ecology communities to grasp, as they require some physical intuition to fully understand. It may be worth adapting the language to describe the mechanisms only in terms of differing rates and in very qualitative terms (e.g., rate of beneficial mutations occurring and subsequently sweeping, rate of neutral turnover of a population, rate of invasion and subsequent takeover of a population). You can then introduce the notion that each rate corresponds to an appropriate timescale in the results section and emphasize that these can be approximately separable processes under certain conditions (or discuss perturbative analysis), simplifying the mathematics and the intuition. I think it’s largely OK as currently written (for me personally), but the readership may be narrowed without this kind of intuitive presentation. As an example highlighting this issue as currently written, on p5 the authors write “In the presence of a diversity maintenance mechanism, enforced with a certain time scale, …”. This language will be misleading; there is a characteristic time scale associated with the rate of generation of new diversity (e.g., the dynamics of mutational influx and establishment during equilibration to mutation-drift balance) and this time scale can be compared to other time scales to determine its relevance (i.e., whether it can be coarse-grained over or treated adiabatically). Nothing here is ‘enforced’, per se.
- The authors need to do a better job pointing to the Methods section while they are discussing results in heuristic terms. The arguments feel somewhat hand-wavy in the Results section but are made very quantitative and clearly in the Methods section (to the point that I only found the calculations more convincing after reading the latter). It would be ideal to present the Methods first because of this, but I understand that this may be due to constraints of the journal format.
- In both the Results and Methods, the authors need to add a number of citations to prior work where the same calculations have been presented previously in the literature. There are places where the authors present results of ‘classical calculations’ without referencing where they come from or under what conditions they are applicable. Equation (1) and the corresponding section in Methods are a good example of these missing references. Plenty of literature is devoted to differentiating this ‘selective sweeps’ regime (i.e., ‘hard’ sweeps where new variation arises on a longer timescale than the establishment + fixation time of a new variant). The establishment rate of new mutations is implicitly used (i.e., $N(t=0) = 1/s$ in the result for equation 1, but no derivation is presented) but not discussed in the paper, nor do the authors cite the appropriate derivation elsewhere (a nice, intuitive presentation of this can be found in https://academic.oup.com/genetics/article/176/3/1759/6062236, for example, in the

case of beneficial mutations). Citations for [20] and [27] in Methods (p17) should also appear in Results (as these were used to derive the expressions appearing in the latter) and citations should be added for the formulae (at least the differential equations) on pages 18—20.

- The authors use two rescalings of time: one in dimensionless units ("steps") and the second rescaled by the timescale for turnover of the metapopulation ("generations"). I find this unnecessarily distracting and confusing to the reader. Please pick one and present all results in those terms; the two definitions are only related by an overall constant, which does not require any additional explanation. Having said that, use of the word "generation" to describe turnover of the metapopulation is inherently confusing to me as a population geneticist and should probably be avoided for clarity. Similarly, use of the symbol s for species rather than the selection coefficient is inherently confusing in the same way; perhaps using sigma for this would be clearest.
- I appreciate that the dynamics the authors are describing are multilayered and relatively complex and thus necessitate various types of coarse graining and simplifying assumptions to avoid overcomplication. However, I am somewhat uncomfortable with the assumptions implicit in treating a single species/lineage as dominant in a subpopulation at any given time. Microbiota have quite large population sizes and, as a result, generically exhibit complex clonal dynamics at any given time. In particular, my baseline assumption would be that the intraspecies dynamics exist in a state of clonal interference rather than in a selective sweeps regime (N is large, mu N is large, N s > 1 for virtually any s, and many mutations simultaneously segregate such that simple selective sweeps are arguably unlikely). This is not even mentioned in the text. See, e.g., https://academic.oup.com/genetics/article/180/4/2163/6063941, https://academic.oup.com/genetics/article/200/2/619/5936186, https://academic.oup.com/genetics/article/192/2/671/5935183 . It is understandable that the authors may prefer not to consider this complexity, however generic, but this implies assumptions about the parameter regimes for N, mu, s, and the associated timescales for both neutral and selective fixation events within each patch. This should at least be discussed in Methods and the Discussion, if only to explain that they focus on a toy model in a specific parameter regime to illustrate the overarching conceptual point.
- I am also a bit uncomfortable that deleterious mutations, which are presumably more frequent than beneficial mutations and perhaps of similar target size to neutral mutations in microbial species due to large N (and thus Ns>1 for tiny s), are not even mentioned. How would their incorporation in the model impact the conclusions? Simulations could be performed to work this out (without requiring analytics), if necessary. Presumably this is somehow wrapped into the definition of the 'diversity maintenance' parameter, but the dynamics are not immediately clear to me because this introduces at least one additional timescale and standing variation containing a distribution of non-negligible fitness effects.
- It took me some time to get comfortable with the numerous terms the authors introduce (e.g., diversity maintenance mechanism, innovation, invasion, migration-sweep, HGT-sweep, etc.). I think the authors have chosen their diction to make their model as generalizable as possible (e.g., to both mutli-species and multi-strain dynamics within a species), but this comes at the cost of defining a host of terms that will invariably confuse the reader. It would be helpful to choose an example and use it to clarify each term. For example, if discussing a single species, innovation is simply related to a large-scale mutational event or enough mutations to generate functional/phenotypic differences. On the other hand, if discussion multiple species, this dynamic is related to speciation. Choosing either one to focus on will clarify all of these concepts and ground the terms that may be too general to be familiar to the readership.
- The authors focus on separation of time scales in the way they present their decomposition into parameter regimes, but it would be particularly helpful to specify and define exactly which time scales they are referring to in the very beginning of Results. This is done much more clearly in the Discussion in the current draft and the appropriate text could be relocated for clarity.
- Various places: the notion of diversity maintenance should somehow be revised to 'maintenance and regeneration of diversity' or something similar. If there is a highly mutative/efficient migratory process at play, much of the lost diversity can be quickly recovered due to the rapid equilibration to mutation-drift or mutation-selection-drift balance after each sweep.

Some specific comments:

- I think it would be helpful to explain why the selection coefficient for beneficial mutations does not need to be explicit in the expressions in Equations (2), (3), etc., and the surrounding text. Intuitively, the selective advantage of a beneficial mutation should dictate the rate of fixation, but this is obscured by various variable rescalings and coarse graining of the intra-species dynamics. Similarly, I had trouble relating the parameters used to point mutational rates; if 'innovation' is a speciation event, this is understandably a distinct variable, but if discussing a metapopulation of demes connected via migration, this should be relatable to the point mutation rate.
- Figures 2C, 3C and D, 4C and E, and S1B should be shown on a log scale (x-axis, or both in some cases) and simulations should be more evenly spaced on a log scale. This would be particularly helpful for the variables defined as ratios (e.g., omega/omega_0). The current plots make it seem like the scale was chosen for agreement between simulations and analytics; instead, showing the breakdown of the analytic approximations would be more instructive, as this helps detail the regime of validity of each approximation. Some of the simulation trends also appear linear (e.g., 4C) simply because of the small scale of the parameter regime plotted.
- Figure 3 caption, second to last sentence: "M=10000 realizations" should not say realizations; last sentence: reference to panels B and C should refer to C and D.
- Page 7: Below Equation 1: "relaxation time to the equilibrium" can be restated everywhere as "equilibration time" (or equilibration time scale). Final paragraph: Here you state that HGT does not reduce diversity (which is true if you count diversity by the number of segregating species in the metacommunity). This is a bit misleading as currently stated, because HGT of a beneficial allele does reduce the intra-population diversity via a sweep; it is only the pan-metagenomic diversity that is maintained. Again, this goes back to the need to clearly define and differentiate metrics of diversity at the beginning of the paper.
- Pages 7-10 (on HGT): Perhaps make it clear that this section assumes only a single beneficial locus and that no two beneficial mutations can simultaneously sweep at distinct loci. I realize this is implicit, but in some sense this is relaxed (and pseudo-accounted for by varying the strength of selection for beneficial innovation events) in the following section on competing time scales. This was only clear to me after reading the latter, so the model seemed oversimplified in this regime. This also explains why Figure 3 does not allow sufficiently large p_h to have multiple simultaneously segregating beneficial events within a patch (which is not accounted for in this construction).
- P10: Equation (3): State clearly what assumptions were made to derive this (e.g., small nu, small p_h). In the following paragraph, it may be worth pointing out that Q_0 ->0 when either p_h vanishes or p_m becomes very large (which have very different biological interpretations).
- There are a lot of parameters to keep track of. Unless I am mistaken, p_m is defined as (1-p_h) until page 12 (on competing time scales). Perhaps just eliminate the variable p_m, and only introduce it in the latter because you want to differentiate the 'nothing happens' event from p_h (1-nu) and p_m (1-nu) 'non-neutral' events. Also, I believe the 'neutral rate ' event (page 12 end of first paragraph) should be (1-nu)(1-p_h - p_m) rather than (1-nu), unless p_m=1-p_h in this section, as well (in which case p_m is fully redundant and can just be removed for clarity, and p_m+p_h = 1, as in the denominator of tau_fix in the following paragraph).
- Page 12, second to last line: "tau" should be "\tau" (Latex typo).
- Page 13: The 'observations' in the first paragraph seem definitional/tautological. Q is defined so that the minimum is Q_0. Following paragraph: omega_0 is not defined and please reference the difference between the left and right insets in Figure 4D.
- Pages 12-13: I find this periodic model of introduction of beneficial alleles highly contrived. I think it may be better to discuss the results for stochastic introduction of beneficial mutations (currently in Fig S1) and mention the cyclical/periodic construction in the supplemental figure. I understand that this was chosen to make the competition between timescales more intuitive, but it does not feel biologically relevant to me.
- Discussion: the model presented here is a coarse graining of complicated metapopulation dynamics, but the fundamental dynamics for each patch and the relation between patches are well understood and can

be simulated explicitly (e.g., using software like SLiM). While you show concordance between your analytics and simulations under the same assumptions, it is not entirely clear to me that this is the only/correct set of emergent dynamics on the metapopulation scale. If you did some sample simulations using a genome-level population genetics simulator (or writing a multiple patch fitness distribution-based simulator with the appropriate parameters), even for M=5-10, it would reinforce the applicability of your model as the correct emergent description. However, I'm very hesitant to suggest you do this because it likely involves a substantial amount of additional work and computational time, but this feels like the right line of support for the kind of *de novo* model building of an effective theory you have presented here, particularly in the absence of clear empirical predictions for specific datasets.

- Discussion: I feel like there is a missing paragraph related to the limitations of this model, including a description of the assumptions/oversimplifications and conjectures on how including each complication (relaxing these simplifications) would alter the results. This also includes the choice of metric for diversity, the number of segregating species.
- Reference 11: the journal 'Science' should be capitalized.

Thank you to both the authors and the editor for the opportunity to review this manuscript. To the authors: I sincerely enjoyed reading your paper and working through the theoretical calculations. My apologies for the lack of brevity, but I wanted to provide you with robust, constructive comments. The majority of my comments should be viewed as (optional) suggestions for improving the text and clarifying the presentation; in my opinion, your analysis is interesting and readable in its current state.
