# Supplementary material for: Metacommunity structure preserves genome diversity in the presence of gene-specific selective sweeps under moderate rates of horizontal gene transfer
Source: PLoS Comput Biol. 2023 Oct 4;19(10):e1011532. doi: 10.1371/journal.pcbi.1011532 (PMC10578598; doi:10.1371/journal.pcbi.1011532)
Supplement: Attachment — Submitted filename: Replies_to_Referees_Subm.pdf [file pcbi.1011532.s009.pdf]

We thank all the four referees for their work on our manuscript. We report below our point-by-point replies to their comments

Reviewer #1:
The Authors present a very nice modeling scenario and analysis to explore how selective sweeps and horizontal gene transfer (HGT) affect diversity in a metacommunity (i.e. spatially structured) setting. The main takeaway of the paper is that spatial structure can preserve diversity by making gene-specific, rather than genomewide, sweeps possible, even for low to moderate rates of HGT. Essentially, this occurs because the sweep rate due to vertical transmission is greatly reduced by the spatial structure; the sweep is forced to occur on the timescale of migration, which allows HGT to be "competitive" with this rate. HGT can then allow significant spread of the beneficial gene in a gene-specific fashion, preserving diversity in the metacommunity. This provides a simple and likely ubiquitous mechanism that can explain the prevalence of gene-specific sweeps inferred from data, which has been difficult to explain given empirical estimates of HGT rates.

The Authors develop a very clean explanation for the phenomenon of interest, and provide an impressive mix of analytical and simulation results. I particularly liked the way the Authors built up the full dynamical picture by first considering a background neutral process, then considering a single sweep event, then finally considering how these processes, occurring on several different timescales, interact. Below I offer some suggestions that could improve the manuscript; I separate these into "Major" and "Minor", although "Minor" and "Very Minor" might be more apt.

We thank the referee for her/his interest in our work.

Major comments
Background and contextualization

(1) I found the paragraph beginning "In the past years" somewhat confusing. First, my reading of the Shapiro et al. paper is that it presents evidence for gene-specific, rather than genomewide sweeps, and also suggests that habitat-specific HGT helps maintain genetic clusters. However, it does not seem to me to provide a hypothesis for the question, "How can gene-specific (or at least not completely genomewide) sweeps occur despite low HGT rates?", which I take to be the central question of the current manuscript. Indeed, Shapiro et al. pose as a major open question "What mechanisms (aside from unrealistically high recombination rates) are responsible for preventing genomewide selective sweeps". Thus, I have a hard time seeing how this constitutes a hypothesized mechanism.

We thank the referee for raising this point. The hypothesis of a "new model of genotypic cluster formation in which an ancestral, ecologically uniform, and recombining population differentiates into novel, ecologically distinct populations, which gradually develop into genotypic clusters" was actually explicitly introduced as a mechanism in

[I] Polz MF, Alm EJ, Hanage WP. Horizontal gene transfer and the evolution of bacterial and archaeal population structure. Trends in Genetics. 2013 Mar 1;29(3):170-5.

and was based on the evidence collected by Shapiro et al. We have now clarified this aspect ad add the citation to [I] in the main text.

(2) Second, the Authors assert that the mechanism proposed by Niehus et al. requires "a small immigration rate and a high HGT rate". What is the basis for this claim? My understanding of the Niehus paper is that diversity is preserved when migration rates are intermediate (in a specific sense) and that the HGT rates they consider are all "low" (at least relative to the strength of selection, and comparable to empirical estimates for HGT). Can the Authors explain a little more here?

The model by Niehus et al. proposes that the combination of (i) HGT rates close to realistic estimates and a (ii) migration rate smaller than the typical selection coefficients of beneficial mutations, can lead to the fixation of beneficial genes without the decrease of genome diversity. However, the model predicts the typical gene sweep to be too fast, and would require high HGT rates (based on current estimates of genetic transfer rates) to match the experimentally observed gene sweep times (as discussed by the authors of Niehus et al in the Discussion section of their paper).

We have added this discussion in the main text, also adding citations to the related literature.

(3) Third, the reference to "basal recombination rate" in the Takeuchi et al. paper is very confusing. The context (basal compared to what?) is not clear here. Additionally, it seems contradictory to say that this hypothesis requires low recombination rates, then state later in the same paragraph that all of these scenarios require high HGT rates. The Authors should clarify each of these three points. I also might suggest moving some of this content to the Discussion, especially if longer exposition is needed to clarify the confusions I mention above.

The basal recombination rate was intended as the spontaneous rate, not affected by selection forces. We have now clarified these aspects in the main text.

(4) Additionally, one major piece of context that seems missing is the classical population genetics perspective on selective sweeps in structured environments. There is a literature on this topic, for example:

Slatkin, M., & Wiehe, T. (1998). Genetic hitch-hiking in a subdivided population. Genetics Research, 71(2), 155-160.
Santiago, E., & Caballero, A. (2005). Variation after a selective sweep in a subdivided population. Genetics, 169(1), 475-483.
Kim, Y., & Maruki, T. (2011). Hitchhiking effect of a beneficial mutation spreading in a subdivided population. Genetics, 189(1), 213-226.

We agree with the reviewer that mentioning this classic view is important. The suggested literature is now cited in the main text.

(5) I believe the present manuscript still contributes plenty of novelty, especially since the questions of interest are somewhat different in the bacterial context, and because the mechanics of HGT are somewhat different than sexual recombination. However, the basic intuition that population structure can increase the diversity preserved through a sweep has been recognized before, e.g. in the references above. It would be good to acknowledge this, and perhaps to clearly identify what is new or different in this context.

We thank the referee for her/his useful and positive comments. Following her/his suggestions, we have now extended our discussion addressing all the raised points (1-4).

Unstructured benchmark
The point that metacommunity structure preserves variation compared to a hard sweep (e.g. a sweep with no HGT) is made clear, but it might be helpful for readers to see an explicit comparison to a scenario without spatial structure. For an unstructured population of size *MN*, how much diversity is preserved? None? My sense is that the resulting sweep would be essentially hard, given the rates considered here, but it would be a nice contrast to explicitly compare against an unstructured benchmark.

While the referee raises an interesting point, in our model the intra-population dynamics is not considered, and, by construction, in each community we only focus on one single species, which can be thought of as the most abundant one present in a given community. Hence, we believe that the proper point of reference, i.e., a null model (benchmark) to compare our result to, is the one where no HGT is not taking place and the spread of species over the metacommunity is attained via migration events only. This is what we have called the "diversity-maintenance process" (Eq (1) + Fig.2), which is now also presented in the main text as a null model for our framework.

Relationship between HGT rates and migration rates
Somewhat related to point above, one aspect of the model framework that confused me a bit was how to think about the relationship between the HGT rate and migration rates. My biological intuition is that they should be linked somehow, such that HGT is inherently less likely when populations are highly separated (e.g. μ_h decreases when μ_m decreases). This is not explicitly enforced, although Eq. 6 implies some relation between these rates. Related to this confusion, what would be the HGT rate in an unstructured population? Should I think of μ_h as the product of μ_m and some baseline HGT rate, which would apply within a local patch? A last point of confusion: The Authors state that this mechanism "is compatible with small HGT rates compared to typical migration time scales", which seems at odds with Eq. 6. Can the Authors clarify all of this?

In our model, HGT and migration rates are independent, and we consider a fully connected network of communities where the spatial distance is not taken into account. We have added this specification in our manuscript. A simple way to explore the case where μ_h decreases when μ_m decreases is the fact that only their ratio matters (Fig. 3C) for the diversity loss in our model, so if they are proportional one would stay within a well-defined stripe of values of their ratio. Clearly, these are just simplifying assumptions in order to define a simple and conceptually clear analytical model, and variants with these ingredients would be interesting to explore in future studies.

As mentioned above (and for similar reasons), the intra-population dynamics is not considered in our model. For the purposes of our investigation, the only requirement we consider is that the fixation dynamics of a beneficial gene is much faster than the migration and HGT dynamics we are investigating, which is the condition enforced by Eq. 6 (based on considerations that are external to the model). The reviewer is correct in saying that the considerations leading to Eq. 6 imply that in order for our approximations to be fully consistent the ratio between μ_h and μ_m cannot become too small or too large. We have specified this point in the revised text.

Probability of extinction

I believe there is a simpler and more accurate way to estimate the probability of extinction (Eq. 21). In the scenario considered here, each patch of a species *s* will meet one of two fates: it will be replaced by a species with the beneficial gene, or it will receive the beneficial gene through HGT. Both events depend on an interaction with a patch possessing the gene already. The rate at which this interaction happens is essentially irrelevant – once a population of species *s* is selected to interact with a population that has the gene (at a total rate $p_h + p_m$), it will be replaced with probability $\frac{p_m}{p_h+p_m}$ and HGT happens otherwise. So, the probability that no population of species *S* survives is just

$$\left(\frac{p_m}{p_h + p_m}\right)^{M_s^0} = \left(1 + \frac{p_h}{p_m}\right)^{-M_s^0}$$

This expression is close to $e^{-M_s^0 \frac{p_h}{p_m}}$ for large M_s^0 and small $\frac{p_h}{p_m}$. One can use this estimate in the sweep parameter, *Q*, with no modification of the derivation of *Q*, and the modified *Q* appears to give a slightly better fit to the simulation data in Fig. 3 (I checked this by eye). Additionally, this argument is much simpler, removing the need for Eqs. 14-20.

We thank the referee for this nice alternative derivation, which actually gives a slightly better approximation of our simulations (see figure below). Although we prefer to not include this derivation in our current manuscript. we will be happy to take it into consideration further development of our model (with a proper acknowledgment of the referee).

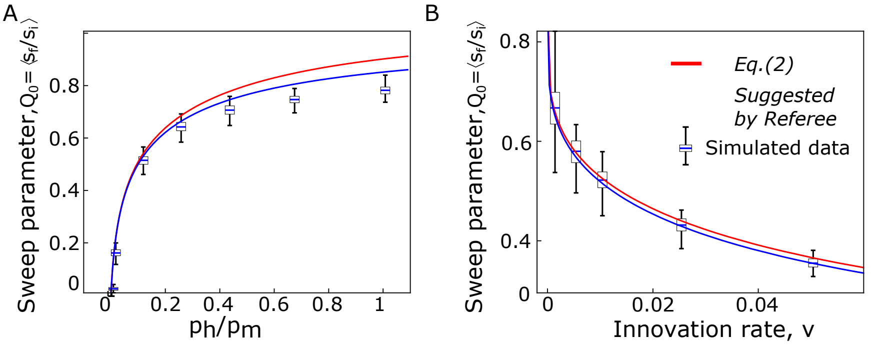

Minor comments

-The Authors provide a very nice picture of the temporal dynamics in their scenario. However, the data needed to see these patterns are rare and difficult to produce. Are there any predictions or diagnostic features that could be observed in, for example, spatial patterns or traditional population genetics data (e.g. site frequency spectrum). I realize this is probably beyond the scope of the Manuscript, so I understand if there is not much to be said here, but I do think that even some discussion on this point would substantially strengthen the manuscript.

We agree with the referee that this is important. We already discussed this aspect in the last paragraph of the Discussion section. We expanded our discussion, although we realize that we are just scratching the surface of this crucial problem.

-The current wording of the title reads awkwardly; I would suggest "A metacommunity scenario preserves diversity in the presence of gene-specific selective sweeps" instead. Additionally, the title seems to mis-state the point slightly – to me as a reader, it's not surprising that gene-specific sweeps preserve diversity, but it is unclear how gene-specific sweeps can compete with genomewide sweeps. Something like "A metacommunity scenario explains gene-specific sweeps under moderate rates of horizontal gene transfer" might be clearer.

We agree with the reviewer, and we have modified the title of the manuscript accordingly.

-The term "diversity-maintenance mechanism" was confusing at first. By this, I believe the Authors mean some mechanism, such as speciation or immigration of new species, that provides a source of new diversity. I appreciate that the Authors want to emphasize that their results are agnostic to a particular assumption about this mechanism, but I would more clearly explain what is meant by this term when it is first used.

We agree that this could create misunderstandings and we have clarified this point in the revised text.

-The use of the term "sweep" for neutral takeover of patches seems likely to cause confusion with selective sweeps. Maybe use an alternate term, such as "neutral takeover" or "drift".

We have modified the terms used for such events in the case of neutral dynamics.

-Instead of "We next ask when diversity decreases only moderately ...", consider a more neutral question, such as "We next ask how much diversity is lost upon the introduction of a beneficial gene, in the absence of diversity-maintenance mechanisms."

We thank the referee for this suggestion. We have rephrased the sentence in the main text.

-The intuition for the scenario described by Eq. 2 is very clear, but the expression itself is not. The genome sweep time has not been introduced yet, so readers would benefit from slightly more explanation here (and maybe a reference to the supplement). Additionally, I am unsure where the second inequality comes from. What is the metacommunity-wide fixation time? Is this the timescale on which all diversity would be lost if neutral takeover is the only process? Please provide slightly more explanation, and clarify where the timescale *MN* comes from here.

The MN timescale accounts for the fixation dynamics of a neutral mutation. In this case, in each patch the time to fixation is of order of the neutral coalescence time N. We clarified this point and added a reference to the Methods section.

-"maintenance" is misspelled as "mantainance" on p. 10.
Fixed.

-Just before Eq. 4 there is a tau missing the backslash.
Fixed.

-I was confused by the statement "Inclusion of a diversity-restoring mechanism [...] can increase the minimal observed diversity as well as the maximal one". Should this say "as well as decrease the maximal one"? Or am I missing something?

We have corrected the sentence.

-Niehus is misspelled both as Niehuis and Niehius at different points. Please double check this.
Fixed.

-Readers might benefit from a reference for the two standard results in the first paragraph of the Methods (the fixation probability and sweep time).

Fixed.

-In the Methods, *s* is used both for the strength of selection and to denote a species. I would use a different letter for the latter purpose, to avoid any confusion.

We have diversified the two definitions: lower case "s" is used for the selection coefficient, while upper case "S" is for the total number of species.

-There is an extra label for Eq. 23.
Fixed.

Reviewer #2:
This paper provides one scenario in which biological populations maintain their diversity through a metacommunity composed of patches against the mechanism of genetic diversity maintenance by neutral mutation and the effect of diversity reduction caused by gene-specific sweeps associated with beneficial horizontal gene transfer (HGT.)

Using numerical simulations and mathematical models, the authors showed that the neutral transfer of individuals or genes among patches and the fixation of beneficial genes could restore diversity, provided that the transfer rate is on a reasonable timescale. The authors argue that this allows diversity to be maintained without assuming high rates of HGT, as claimed in some papers.

The conclusion is not far-fetched and seems reasonable. However, I think it should be added, "Under some assumptions in this study...".

We agree with the reviewer, and we have specified that our model relies on a simplified framework (beginning of the Discussion session).

I have some questions, which I will list below.

(1) the periodicity of the fixation time of the beneficial gene is questionable. In nature, it is acceptable even if the mean of τ_fix is given by a statistic such as Eq. 13, but it seems to occur randomly. So, shouldn't we insist that diversity is not zero in the long-time average?

Eq. 13 is the deterministic limit of a stochastic process (i.e., the full model) which we use only to build an approximated analytic solution of our model. Stochastic fluctuations are indeed in our model. We have now clarified this aspect in the main text.

(2) Is the total number of individuals in this model conserved? The maximum value of B(t) is M, but since D(t) decreases independently, the number of individuals fluctuates, affecting the diversity.

The total number of patches in our model is indeed conserved. The equations 12-14 (and related) are used to derive an approximated solution of the model dynamics (see previous answer), hence should not be confused as the dynamical equations of the model, whose dynamics is investigated with numerical simulations.

(3) is the limit in Eq. 25 consistent with Eq. 2?

We have replaced the limit $M \to \infty$ with the condition M>>1 to avoid confusions and possible misunderstandings when reading Eq. 25 together with Eq. 2.

Minor remarks
P. 12 tau_fix -> τ_fix
Fixed.

P. 20 Delete (23).
Fixed.

Reviewer #3:

This manuscript details a theoretical mechanism intended to address the following counterintuitive observation in microbial metagenomic data: despite a widely held assumption that horizontal gene transfer rates remain small such that the population dynamics are predominantly clonal, there appears to be excessive genetic diversity relative to the expectation that selective sweeps of alleles under positive selection are common in microbiota and efficiently purge variation. Specifically, there is an observed excess of diversity proximate to beneficial alleles (genes), which has been argued in the literature to only happen when horizontal gene transfer (HGT) rates are high. The authors address this issue by postulating that the diversity observed in metagenomic samples is not due to intra-population maintenance (or regeneration via mutations), but instead largely due to population structure. Specifically, they model a large number of relatively isolated subpopulations (connected via migratory dynamics), which are inherently pooled together in a metagenomic sample and collectively sequenced; the observed genetic diversity arises due to demographic diversity of the habitat (many local niches where differing species may rapidly fix), rather than due to intrapopulation dynamics that may require invoking mechanisms like ubiquitous recombination, linkage, epistasis, or unrealistically high mutation rates. The mathematical model the authors specify is thus something closer to an ecological model, rather than a panmictic population genetic model. The authors use both population genetic and eco-evolutionary techniques to analytically describe their constructed system and compare this to simulations, which show reasonable agreement to their theoretical analysis.

Overall, I find their proposition reasonable and largely intuitive. The analytics presented are sound (I rederived all presented formulae to confirm this) and the authors decompose relatively complex dynamics inherent to the system via a separation of timescales argument, which I find both convincing and appealing. I have a number of comments on the presentation of their findings, but, qualitatively, I agree with their results and feel they are sufficiently interesting to warrant eventual publication.

We thank the referee for the positive remarks on our work.

(1) However, I note that there are a substantial number of simplifying assumptions used to construct their toy model, only some of which are discussed in detail in the text; some of these assumptions are not obviously consistent with the majority of microbial metagenomic systems in my view (e.g., microbial subpopulation sizes small enough to result in subsequent selective sweeps, rather than clonal interference; this impacts the assumption that, at any given time, each subpopulation is generically dominated by a only single lineage/species/strain). More substantial text should be dedicated to the regime of applicability of their model (explicitly linking to measurable population genetic and ecological parameters, where possible), and highlighting the effect of each simplifying assumption on the regimes of validity; this should be done in the beginning of the manuscript, rather than the more minor treatment that currently appears in the Discussion. A deeper overview of the nature of the specific types of metagenomic data the authors address, which should appear in the introduction, is also warranted, as this would better frame the problem and make it more obvious that inter-, rather than intra-population dynamics are applicable to the data used in the literature highlighting this outstanding question (e.g., is the initial observation of excessive diversity only seen in pooled short read sequencing studies?; how does ascertainment of a metagenomic sample affect this proposition—if a bacterial metagenomic sample is generated from a minimally invasive biopsy, how does the potential underrepresentation of the metacommunity affect their results?).

We thank the referee for raising this important point. We have extended our discussion concerning the points raised by the referee in the main text. In short, the model predictions concerning the maintenance of the diversity, i.e., whether or not the total diversity is removed during a gene-specific selective sweep, are valid also in the presence intra-population clonal interference effects, since our model provides a lower bound estimate for the total biodiversity of the metacommunity.

(2) Most importantly, the authors should dedicate much more significant real estate on the notion of 'diversity', as the biodiversity-related meaning of diversity in their theoretical study (the number of species in the metapopulation) is entirely different from common measures of genetic diversity in population genetics that may or may not account for allele frequency-based metrics like heterozygosity (i.e., two-point measures of diversity related to the variance of allele frequency, even in haploid species). In my view, genetic diversity is more naturally applicable to pooled short-read metagenomic sequence data, as the ability to correctly separate and identify distinct 'species'/strains is inherently more difficult unless the read data can be mapped to robust genome-wide references. In particular, the expectation of the number of species in a metacommunity uses no allele frequency information; this measure is analogous to population genetic quantification of the number of segregating sites in a sample, which is well known to be substantially influenced/biased by sample size. In contrast, a two-point measure of diversity (analogous to heterozygosity in population genetics) would likely provide a more unbiased estimate of pooled sample 'diversity', and should be similarly straightforward to measure in simulation data and not much more difficult to treat analytically. In the present context, such a measure corresponds to the probability of finding distinct, rather than the same, species when randomly sampling the metapopulation twice, which is related to the variance, rather than the mean, of the distribution of species in the metacommunity. I am not suggesting that the authors should redo their analysis with such a distinct measure, but a discussion of the robustness of their qualitative findings to differing measures of diversity would reinforce their conclusions, particularly if this can be tied back to the varying ways diversity can be measured in metagenomic sample studies.

We have now added a discussion on this aspect, to avoid confusion on the interpretation of the diversity as we intend it in our paper. We have also performed some additional analyses (see Figure S2), which show that that the trend of the variance of the number of species is similar to that of the mean value, and that the variance never goes to zero. This alternative measure is now also presented in the discussion as a possible (far-fetched) link between model and data.

General comments:

- The authors need to do significant copy editing prior to publication. There are many syntactical and grammatical mistakes, along with a few spelling errors.

  We have revised the text addressing this point.

- The introduction highlights a few highly relevant papers but explains their content too briefly. As PLOS Comp. Bio. is an interdisciplinary journal, more detailed presentation of each of these papers would improve the introduction and better frame the problem being addressed. In particular, the mechanisms described in references [6] and [14] are both somewhat realistic, in my view, but are presented very briefly and should be

elaborated on and somewhat clarified in the introduction. The authors should also make it very clear that the rate of recombination discussed in this section is not entirely the same as the rate of HGT (e.g., [14] discusses high HGT rates but low per-base recombination); this was unclear to me during my first read through.

We have expanded the background in our introduction, but we have preferred to leave some details on refs 6 and 14 to the discussion, where the terms of comparison with our model are clearer.

- In my experience, presentation of separation of timescales arguments (e.g., adiabatic approximations) can be difficult for the population genetic/ecology communities to grasp, as they require some physical intuition to fully understand. It may be worth adapting the language to describe the mechanisms only in terms of differing rates and in very qualitative terms (e.g., rate of beneficial mutations occurring and subsequently sweeping, rate of neutral turnover of a population, rate of invasion and subsequent takeover of a population). You can then introduce the notion that each rate corresponds to an appropriate timescale in the results section and emphasize that these can be approximately separable processes under certain conditions (or discuss perturbative analysis), simplifying the mathematics and the intuition. I think it's largely OK as currently written (for me personally), but the readership may be narrowed without this kind of intuitive presentation. As an example highlighting this issue as currently written, on p5 the authors write "In the presence of a diversity maintenance mechanism, enforced with a certain time scale, …". This language will be misleading; there is a characteristic time scale associated with the rate of generation of new diversity (e.g., the dynamics of mutational influx and establishment during equilibration to mutation-drift balance) and this time scale can be compared to other time scales to determine its relevance (i.e., whether it can be coarse-grained over or treated adiabatically). Nothing here is 'enforced', per se.

  We thank the referee for his/her suggestion. Following this advice we have now clarified our language regarding time scale arguments.

- The authors need to do a better job pointing to the Methods section while they are discussing results in heuristic terms. The arguments feel somewhat hand-wavy in the Results section but are made very quantitative and clearly in the Methods section (to the point that I only found the calculations more convincing after reading the latter). It would be ideal to present the Methods first because of this, but I understand that this may be due to constraints of the journal format.

  We thank the referee for his/her suggestion. We have added several references to the Methods in the Results section of the paper.

- In both the Results and Methods, the authors need to add a number of citations to prior work where the same calculations have been presented previously in the literature. There are places where the authors present results of 'classical calculations' without referencing where they come from or under what conditions they are applicable. Equation (1) and the corresponding section in Methods are a good example of these missing references. Plenty of literature is devoted to differentiating this 'selective sweeps' regime (i.e., 'hard' sweeps where new variation arises on a longer timescale than the establishment + fixation time of a new variant). The establishment rate of new mutations is implicitly used (i.e., $N(t=0) = 1/s$ in the result for equation 1, but no derivation is presented) but not discussed in the paper, nor do the authors cite the appropriate derivation elsewhere (a nice, intuitive presentation of this can be found in https://academic.oup.com/genetics/article/176/3/1759/6062236, for example, in the case of beneficial mutations). Citations for [20] and [27] in Methods (p17) should also appear in Results (as these were used to derive the expressions appearing in the latter) and citations should be added for the formulae (at least the differential equations) on pages 18—20.

  We added the suggested references in the Results section. Equations on pages 18-20 were used and derived in our paper as an approximation for our model, hence no reference to the literature is given.

- The authors use two rescalings of time: one in dimensionless units ("steps") and the second rescaled by the timescale for turnover of the metapopulation ("generations"). I find this unnecessarily distracting and confusing to the reader. Please pick one and present all results in those terms; the two definitions are only related by an overall constant, which does not require any additional explanation. Having said that, use of the word "generation" to describe turnover of the metapopulation is inherently confusing to me as a population geneticist and should probably be avoided for clarity. Similarly, use of the symbol s for species rather than the selection coefficient is inherently confusing in the same way; perhaps using sigma for this would be clearest.

We have followed this advice and clarified our language regarding time scale arguments.

- I appreciate that the dynamics the authors are describing are multilayered and relatively complex and thus necessitate various types of coarse graining and simplifying assumptions to avoid overcomplication. However, I am somewhat uncomfortable with the assumptions implicit in treating a single species/lineage as dominant in a subpopulation at any given time. Microbiota have quite large population sizes and, as a result, generically exhibit complex clonal dynamics at any given time. In particular, my baseline assumption would be that the intraspecies dynamics exist in a state of clonal interference rather than in a selective sweeps regime (N is large, mu N is large, N s > 1 for virtually any s, and many mutations simultaneously segregate such that simple selective sweeps are arguably unlikely). This is not even mentioned in the text. See, e.g., https://academic.oup.com/genetics/article/180/4/2163/6063941, https://academic.oup.com/genetics/article/200/2/619/5936186, https://academic.oup.com/genetics/article/192/2/671/5935183 . It is understandable that the authors may prefer not to consider this complexity, however generic, but this implies assumptions about the parameter regimes for N, mu, s, and the associated timescales for both neutral and selective fixation events within each patch. This should at least be discussed in Methods and the Discussion, if only to explain that they focus on a toy model in a specific parameter regime to illustrate the overarching conceptual point.

  We have added a clearer explanation of the assumption, addressing explicitly the regimes of selective sweeps and clonal interference for the single populations. We also discuss mode clearly the consistency of our hypothesis around Eq (6). In terms of how much the low intra-patch diversity can be realistic, this would depend on the system, but while it is clear that microbiota have large population sizes, the population sizes supported by a single patch may vary. It should also be clear that any intra-patch diversity would contribute positively to the diversity under gene-specific sweeps, so that our hypotheses lead us to conservative lower-bound estimates (besides having the advantage of greatly simplifying the problem in terms of relevant parameters).

- I am also a bit uncomfortable that deleterious mutations, which are presumably more frequent than beneficial mutations and perhaps of similar target size to neutral mutations in microbial species due to large N (and thus Ns>1 for tiny s), are not even mentioned. How would their incorporation in the model impact the conclusions? Simulations could be performed to work this out (without requiring analytics), if necessary. Presumably this is somehow wrapped into the definition of the 'diversity maintenance' parameter, but the dynamics are not immediately clear to me because this introduces at least one additional timescale and standing variation containing a distribution of non-negligible fitness effects.

  Although the referee suggests a valid point, we think that a more structured model that properly takes into account all the intra-population (pop gen) dynamics is beyond the scope of the current model, whose aim is to investigate the effect of a meta-population at the inter-population dynamics (see also replies to Referee1).

  As partial answer to the point raised by the referee, we expect deleterious mutations to not have big impact on the dynamics of the number of species in the periodic selection regime, as in that case deleterious mutations would be deleted from the population (their fixation probability being proportional to exp(-N |s|), as predicted by the Kimura fixation probability (see e.g. Ref #30). The clonal interference regime is then much more complex, as many mutations (both deleterious and beneficial) would emerge in the same individual, and hence would be affected by hitch-hiking events (see Ref. #31 and related literature).

  We believe that adding all the aforementioned complexity to our model would deserve a proper investigation in a separate publication.

- It took me some time to get comfortable with the numerous terms the authors introduce (e.g., diversity maintenance mechanism, innovation, invasion, migration-sweep, HGT-sweep, etc.). I think the authors have chosen their diction to make their model as generalizable as possible (e.g., to both mutli-species and multi-strain dynamics within a species), but this comes at the cost of defining a host of terms that will invariably confuse the reader. It would be helpful to choose an example and use it to clarify each term. For example, if discussing a single species, innovation is simply related to a large-scale mutational event or enough mutations to generate functional/phenotypic differences. On the other hand, if discussion multiple species, this dynamic is related to speciation. Choosing either one to focus on will clarify all of these concepts and ground the terms that may be too general to be familiar to the readership.

We have clarified that our model is agnostic, and discussed different specific examples.

- The authors focus on separation of time scales in the way they present their decomposition into parameter regimes, but it would be particularly helpful to specify and define exactly which time scales they are referring to in the very beginning of Results. This is done much more clearly in the Discussion in the current draft and the appropriate text could be relocated for clarity.

  We have followed this advice.

- Various places: the notion of diversity maintenance should somehow be revised to 'maintenance and regeneration of diversity' or something similar. If there is a highly mutative/efficient migratory process at play, much of the lost diversity can be quickly recovered due to the rapid equilibration to mutation-drift or mutation-selection-drift balance after each sweep.

  Agreed and done.

<u>Some specific comments:</u>

- I think it would be helpful to explain why the selection coefficient for beneficial mutations does not need to be explicit in the expressions in Equations (2), (3), etc., and the surrounding text. Intuitively, the selective advantage of a beneficial mutation should dictate the rate of fixation, but this is obscured by various variable rescalings and coarse graining of the intra-species dynamics. Similarly, I had trouble relating the parameters used to point mutational rates; if 'innovation' is a speciation event, this is understandably a distinct variable, but if discussing a metapopulation of demes connected via migration, this should be relatable to the point mutation rate.

  We have added a comment on the selection coefficient

- Figures 2C, 3C and D, 4C and E, and S1B should be shown on a log scale (x-axis, or both in some cases) and simulations should be more evenly spaced on a log scale. This would be particularly helpful for the variables defined as ratios (e.g., omega/omega_0). The current plots make it seem like the scale was chosen for agreement between simulations and analytics; instead, showing the breakdown of the analytic approximations would be more instructive, as this helps detail the regime of validity of each approximation. Some of the simulation trends also appear linear (e.g., 4C) simply because of the small scale of the parameter regime plotted.

  We thank the referee for having provided alternative ways to display our results. We believe that these kind of considerations as well as critical revisions of the published data are of utmost importance for a proper peer-review process. For this reason, we have created a repository containing both the source data as well as the scripts used for the figures:

  https://data.mendeley.com/datasets/w7mdmtrtnp/draft?a=e0031cd7-dfc0-4e8d-b550-d25175c44cd2

- Figure 3 caption, second to last sentence: "M=10000 realizations" should not say realizations; last sentence: reference to panels B and C should refer to C and D.

  Fixed

- Page 7: Below Equation 1: "relaxation time to the equilibrium" can be restated everywhere as "equilibration time" (or equilibration time scale). Final paragraph: Here you state that HGT does not reduce diversity (which is true if you count diversity by the number of segregating species in the metacommunity). This is a bit misleading as currently stated, because HGT of a beneficial allele does reduce the intra-population diversity via a sweep; it is only the pan-metagenomic diversity that is maintained. Again, this goes back to the need to clearly define and differentiate metrics of diversity at the beginning of the paper.

  We have fixed the terminology for the relaxation time and specified that we only deal with community- wide gene pool diversity in our model.

- Pages 7-10 (on HGT): Perhaps make it clear that this section assumes only a single beneficial locus and that no two beneficial mutations can simultaneously sweep at distinct loci. I realize this is implicit, but in some sense this is relaxed (and pseudo-accounted for by varying the strength of selection for beneficial innovation events) in the following section on competing time scales. This was only clear to me after reading the latter, so the model seemed oversimplified in this regime. This also explains why Figure 3 does not allow sufficiently large p_h to have multiple simultaneously segregating beneficial events within a patch (which is not accounted for in this construction).

  Done

- P10: Equation (3): State clearly what assumptions were made to derive this (e.g., small nu, small p_h). In the following paragraph, it may be worth pointing out that Q_0 ->0 when either p_h vanishes or p_m becomes very large (which have very different biological interpretations).

  Done

- There are a lot of parameters to keep track of. Unless I am mistaken, p_m is defined as (1-p_h) until page 12 (on competing time scales). Perhaps just eliminate the variable p_m, and only introduce it in the latter because you want to differentiate the 'nothing happens' event from p_h (1-nu) and p_m (1-nu) 'non-neutral' events. Also, I believe the 'neutral rate ' event (page 12 end of first paragraph) should be (1-nu)(1-p_h - p_m) rather than (1-nu), unless p_m=1-p_h in this section, as well (in which case p_m is fully redundant and can just be removed for clarity, and p_m+p_h = 1, as in the denominator of tau_fix in the following paragraph).

  Since the text is already difficult to follow – we kept to the previous definitions, in order to avoid additional definitions.

- Page 12, second to last line: "tau" should be "\tau" (Latex typo).

  Fixed

- Page 13: The 'observations' in the first paragraph seem definitional/tautological. Q is defined so that the minimum is Q_0. Following paragraph: omega_0 is not defined and please reference the difference between the left and right insets in Figure 4D.

  Fixed

- Pages 12-13: I find this periodic model of introduction of beneficial alleles highly contrived. I think it may be better to discuss the results for stochastic introduction of beneficial mutations (currently in Fig S1) and mention the cyclical/periodic construction in the supplemental figure. I understand that this was chosen to make the competition between timescales more intuitive, but it does not feel biologically relevant to me.

  We decided to keep the narrative as is for simplicity, as in the periodic case the associated time scale makes or results more easily readable.

- Discussion: the model presented here is a coarse graining of complicated metapopulation dynamics, but the fundamental dynamics for each patch and the relation between patches are well understood and can be simulated explicitly (e.g., using software like SLiM). While you show concordance between your analytics and simulations under the same assumptions, it is not entirely clear to me that this is the only/correct set of emergent dynamics on the metapopulation scale. If you did some sample simulations using a genome-level population genetics simulator (or writing a multiple patch fitness distribution-based simulator with the appropriate parameters), even for M=5-10, it would reinforce the applicability of your model as the correct emergent description. However, I'm very hesitant to suggest you do this because it likely involves a substantial amount of additional work and computational time, but this feels like the right line of support for the kind of *de novo* model building of an effective theory you have presented here, particularly in the absence of clear empirical predictions for specific datasets.

  This is an interesting idea which, but we believe it is out of scope. It deserves a future project, and we definitely will consider this line of work.

- Discussion: I feel like there is a missing paragraph related to the limitations of this model, including a description of the assumptions/oversimplifications and conjectures on how including each complication (relaxing these simplifications) would alter the results. This also includes the choice of metric for diversity, the number of segregating species.

  Done

- Reference 11: the journal 'Science' should be capitalized.

  Done

Thank you to both the authors and the editor for the opportunity to review this manuscript. To the authors: I sincerely enjoyed reading your paper and working through the theoretical calculations. My apologies for the lack of brevity, but I wanted to provide you with robust, constructive comments. The majority of my comments should be viewed as (optional) suggestions for improving the text and clarifying the presentation; in my opinion, your analysis is interesting and readable in its current state.

Reviewer #4:

I have reviewed the manuscript "A Metacommunity Scenario Preserves the Diversity in Presence of Gene-specific Selective Sweeps". It explores the context of horizontal evolution in biological communities, indicating how metapopulation dynamics (explicit space) contribute to the maintenance of general biodiversity in communities like bacteria, which generates low rates of genetic variability. The authors employ stochastic dynamics to represent the problem and assume that the chances of migration, innovation, and fixation are low compared to the number of individuals and the system as a whole. In comparison, the authors demonstrate the behavior of neutral dynamics that gradually gains new elements, such as innovation and horizontal gene transfer.

Accordingly, the emergence of a beneficial gene generates strong positive selection pressure, reducing biodiversity by positive selection, while horizontal gene transfer (HGT) of benefic genes to other species/strains contribute to the maintenance of species richness. The authors explore how the rate of new gene generation relative to transfer affects the diversity of the metacommunity context. The simulations demonstrate that the rates of new benefic genes reduce the total biodiversity, while the horizontal transfer of benefic genes contributes to the maintenance of species/strains diversity. Further, it is demonstrated to exist a cyclical behavior of the total number of species when both new strains and HGT interact. By tuning the parameters, it is demonstrated that the total biodiversity is modulated by relative innovation rates.

The article is well-written. The ideas are organized and conveyed clearly and cohesively throughout the text. The authors focused mainly on how low HGT rates are capable of contributing to the maintenance of biodiversity due to interaction with spatial dynamics (migration events). Indeed, the results demonstrate that even low HGT rates produce higher rates of biodiversity than those explained by neutral dynamics or selection, an effect resulting from the metapopulation context. Therefore, the paper presents an interesting proof of concept and plausibility regarding the role of HGT and in the maintenance of species diversity, even under low rates of horizontal transfer, at least from a qualitative point of view.

We thank the referee for the positive feedback on our work.

Despite the relevance of the presented topic and the paper has achieved the main objective, there are a few minor weaknesses.

(1) The article's results have the premise that the rates of emergence of new beneficial strains and their fixation scale with the migration rate. Although the parameters presented by the article connote different phenomena from other models found in the literature, the underlying process has already been explored by multispecies models in related areas. In all cases, conclusions suggest that the effect of spatial dynamics on the maintenance of multispecies populations mediates the antagonistic forces, in terms of rates. Therefore, the model, by itself, represents a new application of stochastic dynamics more than an innovation by itself.

Following this and related comments by Referee#1, we have now extended our discussion of the connections between our model and existing literature in the Introduction section of our paper.

(2) In addition, the boundary conditions of the problem arise doubts concerning the role of HGT to maintain species diversity in more intricate ecological-evolutionary systems. This topic is indeed recognized by authors, and it relativizes whether results achieved by simulations are really applicable to real-world systems.

We have specified that our model makes use some simplifying assumption (see reply to Referee#3). However, despite its simplicity, we believe that some of the model predictions could be tested in targeted experiments (last paragraph of the discussion)

(3) Finally, terms like population and species are sometimes employed in sense of statistical modeling, while sometimes they are interchangeably used in the sense of biology. Because the article deals with both aspects, some passages may lead the most inattentive reader to misinterpret the intended idea. Therefore, I suggest that the authors review the use of these terms throughout the text as a way to provide greater clarity.

We revised the text, discussing in detail the terminology we use and giving arguments for our choice, also following indications from Reviewer 3.
