# Supplementary material for: Metacommunity structure preserves genome diversity in the presence of gene-specific selective sweeps under moderate rates of horizontal gene transfer
Source: PLoS Comput Biol. 2023 Oct 4;19(10):e1011532. doi: 10.1371/journal.pcbi.1011532 (PMC10578598; doi:10.1371/journal.pcbi.1011532)
Supplement: Attachment — Submitted filename: Review_of_revision_to_Pompei2023_PLOSCompBio.pdf [file pcbi.1011532.s010.pdf]

Review of Pompei et al. 2023 (revised submission)
PLOS Computational Biology

I am largely satisfied with the edits the authors have made to address the reviewer comments. In my view, the introduction is substantially improved, many of the confusions arising in the text have been clarified, the added references are appropriate, and the presentation feels overall more robust. I have a few outstanding minor comments.

The authors refer to their model as testable/verifiable at various points, including in the abstract. This mainly refers to the final paragraph of the discussion, which only vaguely describes hypothetical experimental constructs without referencing specific tests that would confirm their analytics. I suggest that the authors specify which of the proposed observables such experiments could record that would potentially differentiate the details of their model from other competing models (number of species as a function of the number of discrete patches, etc.).

The paragraph edited to references limitations of their model appears to only state that they have made simplifying assumptions, relaxation of which may provide the basis for future follow up studies. It seems appropriate to speculate how inclusion of each simplification they mention might alter their qualitative conclusions. It seems appropriate to mention the assumption of neutral-only non-beneficial mutations (i.e., not modeling deleterious mutations which would reduce the overall genetic diversity and potentially alter sweep timescales due to linkage effects). The rate of emergence of novel beneficial mutations nu is assumed to be small and treated as a linearizable quantity; limitation to this perturbative, linear nu regime is appropriate to mention, particularly when discussing beneficial mutations arriving via migration from external demes. The authors did not address my comments about visualization of their plots in logarithmic space; the primary reason to do so is to show the breakdown of the linear analytic regime as quantities such as nu are increased. I agree that inclusion of their simulation data and code in an online repository is appropriate, but showing the breakdown of the regime of validity for their approximations via simulations seems both straightforward and an appropriate part of their theoretical analysis. I will defer to the editor and authors to decide how important doing so is prior to publication.

Minor comments:

A number of copy-editing mistakes remain that need to be corrected prior to publication. I have highlighted are a few examples that I found for the authors convenience. (Note that I am referencing page numbers from the version that includes highlighted edits from the previous draft.). I have also provided a few qualitative suggestions for minor edits that I feel clarify the text.

Author Summary, Page 3: “[…]we find that genome diversity can be preserved under moderate rates”; perhaps change “preserved” to “regenerated”, as was done elsewhere in the manuscript.

Introduction, Page 3: "[…] would require very high recombination rates [11]." Here it seems appropriate to also reference papers that estimate the recombination rates directly from experimental or natural data, as this is essential to the argument that the recombination rates needed are inconsistently high when comparing to observed values.
I am also unsure the phrase "[…] expected values of the recombination rate" is appropriate and sufficiently clear; perhaps replace "expected" with "estimated" and provide appropriate citations. Please also specify what "fast" means when describing the fast decline of HGT rates with genetic distance (e.g., does this imply a specific power law or exponential decay of the rate with genetic distance?; I find the ambiguous comparative language confusing).
Page 4: "Additionally, it requires high HGT rates, as the previous ones." This should be revised along the lines of "Additionally, this requires high HGT rates, as in the previous explanations."
Results, Page 5: "We will further assume that all populations are connected, and we will further assume that migration of beneficial[…]" should be revised to "We will further assume that all populations are connected, that migration of beneficial[…]"
Page 6: "[…](where mu is the mutation rate per […]"; I believe "mutation rate" should be specified to be "beneficial mutation rate" in this context.
Page 8: "[…]which correspond to different combinations"; consider replacing "combinations" with "relative values" such that this appropriately refers to qualitatively distinct parameter regimes.
Typo: "corresponding in this context ti"; "ti" should be "to".
Page 9: "In this regime, the diversity S displays an equilibration dynamics […]" should be "In this regime, the diversity S displays equilibration dynamics[…]".
Page 12: "cand" should be "and".
Page 12-13: "[…] which means that the HGT-sweep rate is ten times slower than the typical migration-sweep time is sufficient […]". This should probably be revised along the lines of "[…] which means that a HGT-sweep rate ten times slower than the typical migration-sweep time is sufficient […]".
Page 15: Discussion of the "maximal diversity". I find these statements less appropriate because the authors have clarified that they are using a conservative model with respect to the minimal diversity. For example, additional complications due to clonal interference (and potentially linkage to deleterious mutations) would likely alter this maximum value. Perhaps I am missing something here, but it seems appropriate to mention that this maximum is likely to not be generalizable beyond the regime of validity of their approximations.
Final paragraph of Results, Page 16: Clonal interference bears mentioning here, as well, in addition to in the introduction.
