# Supplementary material for: Metacommunity structure preserves genome diversity in the presence of gene-specific selective sweeps under moderate rates of horizontal gene transfer
Source: PLoS Comput Biol. 2023 Oct 4;19(10):e1011532. doi: 10.1371/journal.pcbi.1011532 (PMC10578598; doi:10.1371/journal.pcbi.1011532)
Supplement: Attachment — Submitted filename: comments_to_authors_R1.pdf [file pcbi.1011532.s012.pdf]

The Authors have taken several steps to improve this manuscript, and have addressed most of my earlier questions. I believe the basic content of the manuscript is both sound and quite interesting; there remains some room to clarify and streamline the presentation, but overall I have few remaining concerns. I outline some suggestions below for improving the readability and clarity of the manuscript, which are mostly optional but which I recommend the Authors consider. The manuscript also contains some typos and grammatical errors, and could use another careful proof-reading.

Overall: I agree with other reviewers that, in an attempt to keep the language general, the Authors have used terminology that may be confusing to some readers, and can be hard to keep track of. I think this issue has been handled fairly well, but there could be further room to improve, perhaps by committing to a clearer scenario and using relevant terminology (noting the greater generality in the intro or discussion) or some other creative solution. I don't have a strong feeling about this, but recommend that the Authors give it some more careful thought.

Abstract: Should this say "the genome spreads clonally within the community" (not "within the gene")?

Abstract: It would be good to state clearly in the abstract that spatial structure acts together with realistic (moderate) HGT to explain gene-sweeps and high diversity (both HGT and spatial structure are needed).

Abstract: Maybe change "maintained given gene-specific sweeps" to "maintained by gene-specific sweeps".

Introduction, paragraph 2: "in such case" → "in such cases"

Introduction, paragraph 4: It is still somewhat unclear to me how this first mechanism explains the "problem" as described here (i.e. how is diversity maintained when vertical transmission is much more prevalent than horizontal). It is clear that an HGT barrier between populations can prevent the erosion of genotypic clusters, but how does this prevent the replacement of one cluster by another? The key piece seems to be differential selection in different patches, which could maintain diversity by selecting for different species/strains in different locales. If I understand correctly, this aspect (environmental heterogeneity) is very important here and should be emphasized.

Introduction, paragraph 5: In the sentence "However, dynamic models built on this second mechanism predict the typical gene-sweep times to be too fast", it is unclear to me: are predicted sweep times faster or slower than empirical estimates? Please clarify this better.

Introduction, paragraph 6: Maybe add "that previously spread neutrally by HGT onto diverse genetic backgrounds."

Introduction, paragraph 7: More accurate to say "that high recombination rates are necessary for gene-specific selective sweeps."

Model components and terminology, paragraph 1: Do "intra-habitat: and "within-population" mean the same thing here? It is somewhat confusing to use both terms. Maybe the parenthetical comment could read "implying that intra-habitat dynamics are typically characterized by neutral fixation or selective sweep of a single species/strain"

Model components and terminology, paragraph 1: Change “the presence of the beneficial gene on a patch” to “the arrival of a beneficial gene in a patch”

Model components and terminology, paragraph 2: I don’t think N has been defined here…

Model components and terminology, paragraph 2: Clarify “approximation of homogeneous local populations”

Model components and terminology, paragraph 3: Maybe “The metacommunity diversity is quantified …”

Model components and terminology, paragraph 4: This is stated clearly later in the text, but here it may be good to add a sentence after the first one along the lines of “The first mechanism may reduce system-wide diversity, because the species in the invaded patch is replaced by the invader, while in the second scenario the beneficial gene is transferred across genetic backgrounds, with no loss of diversity.”

Model components and terminology, paragraph 4: Maybe clarify here that nu is the rate of **neutral** innovation (beneficial mutations are treated separately, and arise at a different rate)

Dynamics of the diversity of the metacommunity in absence of HGT, paragraph 3: Change “moves” to “events”

Gene-sweep dynamics under competing time scales, paragraph 1: It seems to me that a more natural explanation for the choice of f_0 = D_s/M would be if innovation is due to neutral mutation, which happens on any background (ie species with or without the beneficial gene) with equal likelihood.

Discussion and conclusions, paragraph 1: Re-reading this, it seems incorrect that the diversity-restoring mechanism decreases the maximal diversity. Isn’t this reduction due to a high frequency of beneficial mutations, which is a different mechanism?

Discussion and conclusions, paragraph 4: It is very unclear to me how this mechanism leads to an effective frequency-dependent selection. The Authors should explain this assertion much better; I really have no idea what this refers to.
