# Supplementary material for: Metacommunity structure preserves genome diversity in the presence of gene-specific selective sweeps under moderate rates of horizontal gene transfer
Source: PLoS Comput Biol. 2023 Oct 4;19(10):e1011532. doi: 10.1371/journal.pcbi.1011532 (PMC10578598; doi:10.1371/journal.pcbi.1011532)
Supplement: Attachment — Submitted filename: Replies_to_Referees2.pdf [file pcbi.1011532.s013.pdf]

We thank the editor and all the four referees for their work on our manuscript. We report below our point-by-point replies to their comments

Reviewer #1:
I am largely satisfied with the edits the authors have made to address the reviewer comments. In my view, the introduction is substantially improved, many of the confusions arising in the text have been clarified, the added references are appropriate, and the presentation feels overall more robust. I have a few outstanding minor comments.

**1)** The authors refer to their model as testable/verifiable at various points, including in the abstract. This mainly refers to the final paragraph of the discussion, which only vaguely describes hypothetical experimental constructs without referencing specific tests that would confirm their analytics. I suggest that the authors specify which of the proposed observables such experiments could record that would potentially differentiate the details of their model from other competing models (number of species as a function of the number of discrete patches, etc.).

We are grateful for the referee's input. We think that the main things to focus on would involve being able to detect the presence of selective forces at play in genomic regions beyond the one where the favored gene is located. We have now clarified this point in our discussion.

**2)** The paragraph edited to references limitations of their model appears to only state that they have made simplifying assumptions, relaxation of which may provide the basis for future follow up studies.

-It seems appropriate to speculate how inclusion of each simplification they mention might alter their qualitative conclusions. It seems appropriate to mention the assumption of neutral-only non-beneficial mutations (i.e., not modeling deleterious mutations which would reduce the overall genetic diversity and potentially alter sweep timescales due to linkage effects).

We have added a paragraph in the discussion, addressing this point.

-The rate of emergence of novel beneficial mutations nu is assumed to be small and treated as a linearizable quantity; limitation to this perturbative, linear nu regime is appropriate to mention, particularly when discussing beneficial mutations arriving via migration from external demes. The authors did not address my comments about visualization of their plots in logarithmic space; the primary reason to do so is to show the breakdown of the linear analytic regime as quantities such as nu are increased. I agree that inclusion of their simulation data and code in an online repository is appropriate, but showing the breakdown of the regime of validity for their approximations via simulations seems both straightforward and an appropriate part of their theoretical analysis. I will defer to the editor and authors to decide how important doing so is prior to publication.

We thank the referee for having clarified her/his remark. We have now added a supplementary figure (Fig.S3, see below) to show the comparison of our analytical results to simulated data using a log-x scale, for data previously shown in Fig. 2C, Fig.3C and Fig 3D. Panels of Fig.4 were not included in this figure, as no analytical solution was derived in our manuscript.

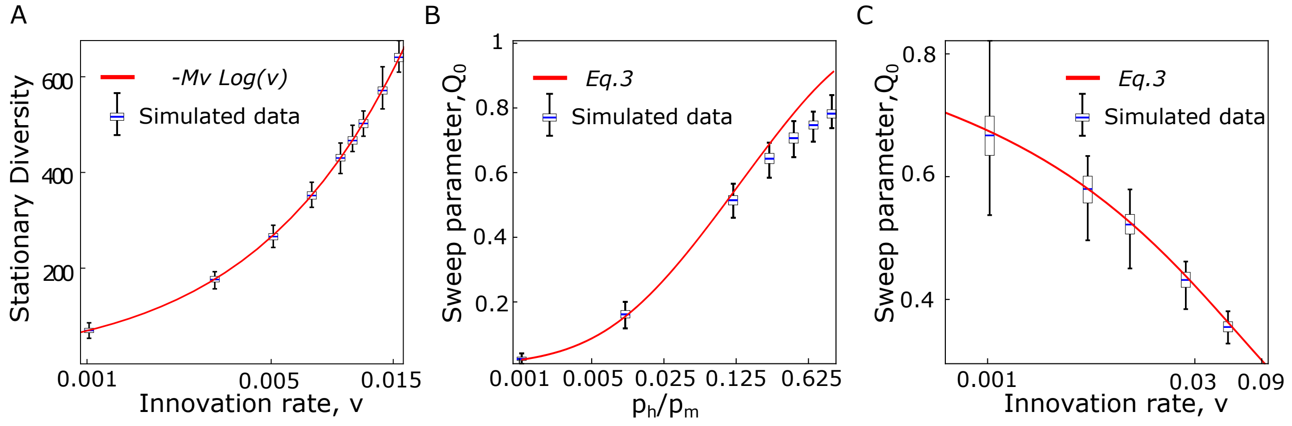


**Minor comments:**

A number of copy-editing mistakes remain that need to be corrected prior to publication. I have highlighted are a few examples that I found for the authors convenience. (Note that I am referencing page numbers from the version that

includes highlighted edits from the previous draft.). I have also provided a few qualitative suggestions for minor edits that I feel clarify the text.

Author Summary, Page 3: "[…]we find that genome diversity can be preserved under moderate rates"; perhaps change "preserved" to "regenerated", as was done elsewhere in the manuscript.
Done.

Introduction, Page 3: "[…] would require very high recombination rates [11]." Here it seems appropriate to also reference papers that estimate the recombination rates directly from experimental or natural data, as this is essential to the argument that the recombination rates needed are inconsistently high when comparing to observed values.
Done.

I am also unsure the phrase "[…] expected values of the recombination rate" is appropriate and sufficiently clear; perhaps replace "expected" with "estimated" and provide appropriate citations.
Done.

Please also specify what "fast" means when describing the fast decline of HGT rates with genetic distance (e.g., does this imply a specific power law or exponential decay of the rate with genetic distance?; I find the ambiguous comparative language confusing).

Done.

Page 4: "Additionally, it requires high HGT rates, as the previous ones." This should be revised along the lines of "Additionally, this requires high HGT rates, as in the previous explanations."
Done.

Results, Page 5: "We will further assume that all populations are connected, and we will further assume that migration of beneficial[…]" should be revised to "We will further assume that all populations are connected, that migration of beneficial[…]"
Done.

Page 6: "[…](where mu is the mutation rate per […]"; I believe "mutation rate" should be specified to be "beneficial mutation rate" in this context.

Changed.

Page 8: "[…]which correspond to different combinations"; consider replacing "combinations" with "relative values" such that this appropriately refers to qualitatively distinct parameter regimes.
Done.

Typo: "corresponding in this context ti"; "ti" should be "to".
Done.

Page 9: "In this regime, the diversity S displays an equilibration dynamics […]" should be "In this regime, the diversity S displays equilibration dynamics[…]".
Done.

Page 12: "cand" should be "and".
Done.

Page 12-13: "[…] which means that the HGT-sweep rate is ten times slower than the typical migration-sweep time is sufficient […]". This should probably be revised along the lines of "[…] which means that a HGT-sweep rate ten times slower than the typical migration-sweep time is sufficient […]".
Done.

Page 15: Discussion of the "maximal diversity". I find these statements less appropriate because the authors have clarified that they are using a conservative model with respect to the minimal diversity. For example, additional complications due to clonal interference (and potentially linkage to deleterious mutations) would likely alter this maximum value. Perhaps I am missing something here, but it seems appropriate to mention that this maximum is likely to not be generalizable beyond the regime of validity of their approximations.

We now specify that we are discussing the maximal diversity observed specifically in our model.

Final paragraph of Results, Page 16: Clonal interference bears mentioning here, as well, in addition to in the introduction.
Done.

Reviewer 2:

The Authors have taken several steps to improve this manuscript, and have addressed most of my earlier questions. I believe the basic content of the manuscript is both sound and quite interesting; there remains some room to clarify and streamline the presentation, but overall I have few remaining concerns. I outline some suggestions below for improving the readability and clarity of the manuscript, which are mostly optional but which I recommend the Authors consider. The manuscript also contains some typos and grammatical errors, and could use another careful proof-reading.

Overall: I agree with other reviewers that, in an attempt to keep the language general, the Authors have used terminology that may be confusing to some readers, and can be hard to keep track of. I think this issue has been handled fairly well, but there could be further room to improve, perhaps by committing to a clearer scenario and using relevant terminology (noting the greater generality in the intro or discussion) or some other creative solution. I don't have a strong feeling about this, but recommend that the Authors give it some more careful thought.

We can see the point of the reviewer, but at this stage such a change would require a global restructuring of the text, which we are afraid could do more damage than good. Nevertheless, we went through the text once more to locally add some explanations and clarifications about the terminology and its meaning.

Abstract: Should this say "the genome spreads clonally within the community" (not "within the gene")?
Changed to "whereby the genome spreads clonally within the community and together with the beneficial gene"

Abstract: It would be good to state clearly in the abstract that spatial structure acts together with realistic (moderate) HGT to explain gene-sweeps and high diversity (both HGT and spatial structure are needed).
Done

Abstract: Maybe change "maintained given gene-specific sweeps" to "maintained by gene-specific sweeps".
Done

Introduction, paragraph 2: "in such case" → "in such cases"
Done

Introduction, paragraph 4: It is still somewhat unclear to me how this first mechanism explains the "problem" as described here (i.e. how is diversity maintained when vertical transmission is much more prevalent than horizontal). It is clear that an HGT barrier between populations can prevent the erosion of genotypic clusters, but how does this prevent the replacement of one cluster by another? The key piece seems to be differential selection in different patches, which could maintain diversity by selecting for different species/strains in different locales. If I understand correctly, this aspect (environmental heterogeneity) is very important here and should be emphasized.

We have clarified this aspect.

Introduction, paragraph 5: In the sentence "However, dynamic models built on this second mechanism predict the typical gene-sweep times to be too fast", it is unclear to me: are predicted sweep times faster or slower than empirical estimates? Please clarify this better.
The sweep times would be too short when compared to current estimates. We have clarified this aspect in the text.

Introduction, paragraph 6: Maybe add "that previously spread neutrally by HGT onto diverse genetic backgrounds."
Done

Introduction, paragraph 7: More accurate to say "that high recombination rates are necessary for genespecific selective sweeps."
Changed

Model components and terminology, paragraph 1: Do “intra-habitat: and “within-population” mean the same thing here? It is somewhat confusing to use both terms. Maybe the parenthetical comment could read “implying that intra-habitat dynamics are typically characterized by neutral fixation or selective sweep of a single species/strain”
Changed.

Model components and terminology, paragraph 1: Change “the presence of the beneficial gene on a patch” to “the arrival of a beneficial gene in a patch”
Changed

Model components and terminology, paragraph 2: I don’t think N has been defined here…
Definition added

Model components and terminology, paragraph 2: Clarify “approximation of homogeneous local populations”
Clarified. We meant to say the “assumption of phenotypically homogeneous populations”

Model components and terminology, paragraph 3: Maybe “The metacommunity diversity is quantified …”
Changed

Model components and terminology, paragraph 4: This is stated clearly later in the text, but here it may be good to add a sentence after the first one along the lines of “The first mechanism may reduce system-wide diversity, because the species in the invaded patch is replaced by the invader, while in the second scenario the beneficial gene is transferred across genetic backgrounds, with no loss of diversity.”
Changed

Model components and terminology, paragraph 4: Maybe clarify here that nu is the rate of neutral innovation (beneficial mutations are treated separately, and arise at a different rate)
Clarified

Dynamics of the diversity of the metacommunity in absence of HGT, paragraph 3: Change “moves” to “events”
Changed

Gene-sweep dynamics under competing time scales, paragraph 1: It seems to me that a more natural explanation for the choice of f_0 = D_s/M would be if innovation is due to neutral mutation, which happens on any background (ie species with or without the beneficial gene) with equal likelihood.
We thank the referee for his/her suggestion. We agree and have added this explanation in our manuscript.

Discussion and conclusions, paragraph 1: Re-reading this, it seems incorrect that the diversity-restoring mechanism decreases the maximal diversity. Isn’t this reduction due to a high frequency of beneficial mutations, which is a different mechanism?
We agree and have clarified this aspect in our manuscript.

Discussion and conclusions, paragraph 4: It is very unclear to me how this mechanism leads to an effective frequency-dependent selection. The Authors should explain this assertion much better; I really have no idea what this refers to.

We mean that the mechanism described leads to the same effect of the frequency-dependent selection, introduced by Takeuchi and coworkers. We have now clarified this aspect in the revised manuscript text.
