# Supplementary material for: Metacommunity structure preserves genome diversity in the presence of gene-specific selective sweeps under moderate rates of horizontal gene transfer
Source: PLoS Comput Biol. 2023 Oct 4;19(10):e1011532. doi: 10.1371/journal.pcbi.1011532 (PMC10578598; doi:10.1371/journal.pcbi.1011532)
Supplement: Attachment — Submitted filename: comments.docx [file pcbi.1011532.s006.docx]

This paper provides one scenario in which biological populations maintain their diversity through a metacommunity composed of patches against the mechanism of genetic diversity maintenance by neutral mutation and the effect of diversity reduction caused by gene-specific sweeps associated with beneficial horizontal gene transfer (HGT.)

Using numerical simulations and mathematical models, the authors showed that the neutral transfer of individuals or genes among patches and the fixation of beneficial genes could restore diversity, provided that the transfer rate is on a reasonable timescale.　The authors argue that this allows diversity to be maintained without assuming high rates of HGT, as claimed in some papers.

The conclusion is not far-fetched and seems reasonable. However, I think it should be added, "Under some assumptions in this study...".

I have some questions, which I will list below.

1. the periodicity of the fixation time of the beneficial gene is questionable. In nature, it is acceptable even if the mean of τ_fix is given by a statistic such as Eq. 13, but it seems to occur randomly. So, shouldn't we insist that diversity is not zero in the long-time average?
2. Is the total number of individuals in this model conserved?　The maximum value of B(t) is M, but since D(t) decreases independently, the number of individuals fluctuates, affecting the diversity.
3. 3. is the limit in Eq. 25 consistent with Eq. 2?

Minor remarks

P. 12 tau_fix -> τ_fix

P. 20 Delete (23).
